# A nanoscale DNA force spectrometer capable of applying tension and compression on biomolecules

**DOI:** 10.1093/nar/gkab656

**Published:** 2021-08-06

**Authors:** Yuchen Wang, Jenny V Le, Kyle Crocker, Michael A Darcy, Patrick D Halley, Dengke Zhao, Nick Andrioff, Cassie Croy, Michael G Poirier, Ralf Bundschuh, Carlos E Castro

**Affiliations:** Department of Mechanical and Aerospace Engineering, The Ohio State University, Columbus, OH 43210, USA; Department of Mechanical and Aerospace Engineering, The Ohio State University, Columbus, OH 43210, USA; Biophysics Graduate Program, The Ohio State University, Columbus, OH 43210, USA; Department of Physics, The Ohio State University, Columbus, OH 43210, USA; Department of Physics, The Ohio State University, Columbus, OH 43210, USA; Department of Mechanical and Aerospace Engineering, The Ohio State University, Columbus, OH 43210, USA; Department of Physics, The Ohio State University, Columbus, OH 43210, USA; Department of Chemical and Biomolecular Engineering, The Ohio State University, Columbus, OH 43210, USA; Department of Mechanical and Aerospace Engineering, The Ohio State University, Columbus, OH 43210, USA; Biophysics Graduate Program, The Ohio State University, Columbus, OH 43210, USA; Department of Physics, The Ohio State University, Columbus, OH 43210, USA; Department of Chemistry and Biochemistry, The Ohio State University, Columbus, OH 43210, USA; Biophysics Graduate Program, The Ohio State University, Columbus, OH 43210, USA; Department of Physics, The Ohio State University, Columbus, OH 43210, USA; Department of Chemistry and Biochemistry, The Ohio State University, Columbus, OH 43210, USA; Division of Hematology, Department of Internal Medicine, The Ohio State University, Columbus, OH 43210, USA; Department of Mechanical and Aerospace Engineering, The Ohio State University, Columbus, OH 43210, USA; Biophysics Graduate Program, The Ohio State University, Columbus, OH 43210, USA

## Abstract

Single molecule force spectroscopy is a powerful approach to probe the structure, conformational changes, and kinetic properties of biological and synthetic macromolecules. However, common approaches to apply forces to biomolecules require expensive and cumbersome equipment and relatively large probes such as beads or cantilevers, which limits their use for many environments and makes integrating with other methods challenging. Furthermore, existing methods have key limitations such as an inability to apply compressive forces on single molecules. We report a nanoscale DNA force spectrometer (nDFS), which is based on a DNA origami hinge with tunable mechanical and dynamic properties. The angular free energy landscape of the nDFS can be engineered across a wide range through substitution of less than 5% of the strand components. We further incorporate a removable strut that enables reversible toggling of the nDFS between open and closed states to allow for actuated application of tensile and compressive forces. We demonstrate the ability to apply compressive forces by inducing a large bend in a 249bp DNA molecule, and tensile forces by inducing DNA unwrapping of a nucleosome sample. These results establish a versatile tool for force spectroscopy and robust methods for designing nanoscale mechanical devices with tunable force application.

## INTRODUCTION

Molecular force spectroscopy has been widely used to probe the structure, conformational changes, stability, and kinetics of biomolecules and molecular complexes. The most common methods, including optical tweezers, magnetic tweezers and atomic force microscopy (1–3), have been used to study a variety of biomolecules such as titin (4), motor proteins (5), signaling proteins (6) and nucleic acids (7) under tension. While force spectroscopy methods have become well-established, there remain key challenges to broadening their application. Specifically, applying forces to biomolecules typically requires specialized equipment and the use of relatively large probes (e.g. microbeads or cantilevers), which limits the ability to perform force spectroscopy in many environments and makes integrating with other methodologies challenging. Recently developed techniques such as centrifugal force microscopy and acoustic force spectroscopy address some limitations, for example by increasing throughput (8,9); but both still require specialized equipment and the use of large probes (i.e. microbeads). In general, mechanical loading on biomolecules can take different forms such as tension, compression, torque, and shear. All of the above-mentioned techniques have been used to apply tension to biological samples. Magnetic tweezers or optical tweezers have been used to apply torque to long macromolecules (10,11). Atomic force microscopy or magnetic tweezers have been used to apply compression or shear on cells or other larger samples (12,13). However, the need for long handles to attach small molecules or molecular complexes to probes (e.g. beads or cantilevers) or surfaces has prevented the exertion of compression forces on such molecular sized samples with current techniques. Here, we seek to address these challenges through the development of a reconfigurable nanomechanical DNA origami device that can apply tunable forces, including compression, to biomolecules.

DNA origami structures (14–16), are uniquely suited to engineer mechanical testing devices at molecular length scales due to their precise nanoscale geometry, programmable mechanical and dynamic properties, actuated reconfiguration, low cost, and versatile interfacing with other molecules (17–19). Many studies have previously reported nanodevices to study biomolecules (20), including hinge or caliper designs to probe or control biomolecules (21–23). In some cases, the devices have been used to apply a force due to the energy cost associated with adopting a particular state of the device when a molecule is incorporated or an interaction forms (22,23). For example, latching a hinge device that tends to exhibit relatively large angles into a closed state via binding of a molecular complex between the arms creates a tensile force on that complex. Other efforts have leveraged the entropic elasticity of single-stranded DNA (ssDNA) components to apply forces (24,25). While these devices have demonstrated utility for probing biomolecules, key capabilities are still missing to enable versatile tools for force spectroscopy including: a single device applying multiple forces; applying different loading conditions (e.g. tension and compression); and triggered force application. A nanodevice with these capabilities could provide a unique approach to study physical properties of biomolecules and molecular complexes.

Here we develop a nanoscale DNA Force Spectrometer (nDFS) that enables tunable force application. First, we demonstrate an approach to engineer the nDFS free energy landscape across a wide range of conformational distributions to tune the free energy cost to adopt specific states. Second, we introduce an approach for direct force application through actuated reconfiguration of the nDFS, which relies on a removable strut to toggle the device between open and closed states. We demonstrate the capability to apply forces by compressing a sample DNA molecule to induce a strongly bent state, similar to a post-buckling configuration of a slender beam, and the ability to apply tension by inducing varying degrees of unwrapping of a nucleosome sample. These results establish a versatile tool for force spectroscopy and robust methods for designing mechanical devices with passive and active force application.

## MATERIALS AND METHODS

### Preparation of the DNA Origami nDFS

The nDFS device was designed using the software caDNAno (26) with an 8064 nt ssDNA scaffold (15) (M13MP18 based scaffold prepared in our laboratory as described in (27)). The staple strand sequences were generated and sub-divided based on the modules of the nDFS (e.g. top arm, struts, hinge vertex, etc.). The caDNAno design is depicted in Supplementary Figure S1. The staples were ordered from a commercial vendor (IDT, Coralville, IA, Supplementary Tables S1–S3). Prior to folding, staples were selected based on the desired device design.

Folding reactions were carried out using previously described protocols (28). Briefly, the reactions contained 20 nM scaffold and 200 nM of each staple strand in a ddH_2_O solution containing 5 mM Tris, 5 mM NaCl, 1 mM EDTA and 18 mM MgCl_2_, at pH 8.0. An initial test of MgCl_2_ concentrations revealed 18 mM yielded high quality folding (Supplementary Figure S2). This folding reaction was subjected to thermal annealing in a thermal cycler (Bio-Rad, Hercules, CA) consisting of rapidly heating the solution to 70°C for 15 min, followed by annealing over the range of 63–57°C for 3 hours per degree Celsius, and then cooling for 30 min at 4°C.

### Purification of the nDFS

The structures were subjected to agarose gel electrophoresis or purified by centrifugation in a polyethylene glycol (PEG) solution (29). Gel electrophoresis was carried out by running structures in a 2% agarose gel prepared with 0.5× TBE containing net 10 mM MgCl_2_ and pre-stained with 1.2 μM EtBr. Gels were run at 70 V for 90 min in a gel running buffer of 0.5× TBE with net 10mM MgCl_2_. For gel purification, bands with well-folded structures were excised from the gel, and structures were recovered by centrifuging at 10 000 g for 10 min in freeze-and-squeeze gel extraction spin columns (Biorad, Hercules, CA). The cut band typically yielded 0.5–2 nM of the structure, which was directly used for imaging by Transmission Electron Microscopy (TEM). Alternatively, centrifugal purification in the presence of PEG was performed by mixing PEG buffer (15% PEG MW8000, 200 mM NaCl and 100 mM Tris) with an equal volume of folded DNA origami structures followed by 16,000 g centrifugation for 30 min. Structures were then resuspended in 0.5× TBE with net 10mM MgCl_2_ to a concentration of 20 nM as quantified by measuring UV absorption on a NanoDrop (NanoDrop 2000C Spectrophotometer, Thermo Scientific). The structures were diluted to 1 nM (using the same buffer, 0.5× TBE with net 10mM MgCl_2_) for TEM imaging.

### TEM imaging and image analysis

For TEM grid preparation, 4 μl of sample volume was deposited on Formvar-coated copper TEM grids, stabilized with evaporated carbon film (Ted Pella; Redding, CA). The sample was incubated on the grid for 4 min and then wicked away with filter paper. The sample was then stained by applying 10 μl 2% uranyl formate (SPI, West Chester, PA) twice for 2 and 15 s, respectively, and stain solution was wicked away after each incubation with filter paper. TEM imaging was carried out at the OSU Campus Microscopy and Imaging Facility on an FEI Tecnai G2 Spirit TEM at an acceleration voltage of 80 kV at a magnification of 45,000×.

The raw TEM images (Supplementary Figure S3) were first organized into galleries (Supplementary Figure S4) using the EMAN2 software particle picking feature, which streamlined the angle measurement process. Angles were measured manually using the software ImageJ by drawing two straight lines directly on the particle image along the inner edges of the hinge arms.

We used MATLAB as the post-processing tool to convert the angle data sets to probability density histograms. Experimental results revealed that the angle distribution plots converged well when the sample size was greater than around 150. The purification and characterization of the nDFS was conducted in duplicate or triplicate (Supplementary Table S4).

### Torque analysis of nDFS devices

The angular free energy landscape was calculated assuming a Boltzmann distribution (30). The free energy landscape was fit to a smoothed spline for visualization purposes. The spline fit was cut off at the edges where we observed relatively few conformations (probability density < 0.001). All free energy landscapes were set to zero at the minimum value; hence, the curves show the changes in free energy relative to the minimum, which is our primary interest.

To estimate the forces applied by the nDFS devices, we first calculated the torque versus angle behavior of each device by differentiating the free energy landscape. This was done by numerically differentiating the spline fit to the free energy landscape data points. The force was then calculated for each position along the hinge arms by dividing the torque by the radial position coordinate. To quantify the uncertainty in the free energy, torque, and force values, we used a bootstrapping approach by re-sampling the angular distribution with replacement (e.g. an angular distribution with *N* = 500 values was randomly sampled 500 times with replacement). This sampling was repeated 50 times to calculate a mean and standard deviation of the parameter of interest (e.g. force at a particular conformation), and the standard deviation was used as the parameter uncertainty.

### Internal strut toggling

To toggle the nDFS open, devices folded in the closed state at 1 nM were incubated with opening strands at a concentration of 125 nM for 1 h at 37°C in 0.5× TBE solution containing 10 mM MgCl_2_. The actuation from the Toggled Open state to the Toggled Closed state was carried out by mixing Toggled Open devices at 1 nM with 1 nM closing strands for 1 h at 37°C also in 0.5× TBE with 10 mM MgCl_2_. The specific strand concentration was chosen to maximize the toggling efficiency at the 1 h time point. When performing successive actuation (i.e. toggling open after toggling closed), centrifugal purification in the presence of PEG was conducted three times after nDFS was toggled closed to remove excess opening strands, ensuring closing strands can properly bind to the nDFS without competition from free strands present in solution.

### Applying compressive force through actuated conformational change

The 249 bp biotinylated dsDNA (Supplementary Table S5) was prepared by PCR using oligonucleotides containing a biotin label on the 5′ end and a pUC19 plasmid template. PCR-synthesized DNA molecules were purified by an anion-exchange HPLC using a Gen-Pak Fax column (Waters). Purified, biotinylated DNA was then mixed with labeled neutravidin (Thermo Fisher A6378) in a 1:10 molar ratio, allowed to incubate at room temperature for 30 min, and then purified by sucrose gradient as previously described (22).

Separately, the biotinylated nDFS was directly folded into an open state by using 30-fold excess of opening strands relative to scaffold during the annealing process. The devices were purified by centrifugation in the presence of PEG as previously described. The 249 bp dsDNA was diluted in nDFS buffer (0.5 TBE, 10 mM net MgCl_2_) and mixed with the purified Folded Open nDFS (nDFS at 2 nM) with the dsDNA sample at 3-fold excess (this ratio gives 62% binding efficiency, optimization test in Supplementary Figure S5) for 30 min at 37°C, and 250 rpm on a temperature controlled shaker (Fisher Scientific isotemp 270600f). After incorporating the 249 bp dsDNA sample, the nDFS-dsDNA system was toggled closed by using closing strands following the same protocol described in the Internal Strut Toggling section.

### DNA compression prediction model

In the dsDNA compression demonstration, the model used to predict the angle distribution of the hinge-dsDNA system has three components: (i) the nDFS, (ii) the flexible connection region at the arm tip and (iii) the dsDNA linker (Supplementary Figure S6). The flexible connection consists of 6-base ssDNA overhangs, two biotins and neutravidin, which are lumped together and modeled as a Gaussian polymer. The probability distribution of the end-to-end distance ${R_p}$ of a Gaussian polymer in three-dimensional space is given by (31):

(1) $$\begin{equation*} p( R_p ) = \left( \frac{3}{2 \pi n{b^2}}\right)^{3/2} \exp \left( - \frac{{3{R_p^2}}}{{2n{b^2}}} \right) \end{equation*}$$

where $n$ is the number of segments in the Gaussian polymer, and $b$ is the Kuhn length of each segment.

The dsDNA linker is modeled as a wormlike chain. Since in the experiment the linker is relatively short (contour length ${L_c}$ = 85 nm, and thus not much longer than the persistence length of dsDNA ${l_P} =$ 50 nm (32–34)), we apply Frey's formula (35)

(2) $$\begin{equation*}{p_{dsDNA}}\left( {{R_D}} \right) \approx \frac{{{l_p}}}{{N{L_c^2}}}f\left( {\frac{{{l_p}}}{{{L_c}}}\left( {1 - \frac{{{R_D}}}{{{L_c}}}} \right)} \right)\end{equation*}$$

Where

(3) $$\begin{eqnarray*}&&\!\!\!f\left( x \right)\nonumber\\ &&\!\!\!= \left\{ \begin{array}{@{}c@{}} {\displaystyle\frac{\pi }{2}\left[ {\exp ( - {\pi ^2}x} ) - 4\exp ( - 4{\pi ^2}x){\rm{ + 9\;exp}}( {{\rm{ - 9}}{\pi ^2}x} )\right]}\\ \ \ \ \ \ \ \ \ \ \ \ \ \ \ \ \ \ \ \ \ \ \ \ \ \ \ \ \ \ \ \ \ \ \ \ \ \ \ \ \ \ \ \ \ \ \ \ \ \ \ \ \ \ \ \ \ \ \ \ \ \ \ \ \ \ \ \ \ \ \ \ \ \ \ \ \ \ {\rm{\;for\;x > 0}}{\rm{.2}}\\ {\displaystyle\frac{1}{{8{{(\pi x)}^{3/2}}}}\left[ \left( {\displaystyle\frac{1}{x} - 2} \right)\exp \left( - \displaystyle\frac{1}{{4x}} \right) + \left( {\displaystyle\frac{9}{x}{\rm{ - 2}}} \right)\exp \left( - \displaystyle\frac{9}{{4x}}\right) + \left( {\displaystyle\frac{{25}}{x}{\rm{ - 2}}} \right){\rm{exp}}\left( { - \displaystyle\frac{{25}}{{4x}}} \right)\right]}\\ \ \ \ \ \ \ \ \ \ \ \ \ \ \ \ \ \ \ \ \ \ \ \ \ \ \ \ \ \ \ \ \ \ \ \ \ \ \ \ \ \ \ \ \ \ \ \ \ \ \ \ \ \ \ \ \ \ \ \ \ \ \ \ \ \ \ \ \ \ \ \ \ \ \ \ \ \ \ \ \ \ \ \ \ \ \ \ \ \ \ \ \ \ \ \ \ \ \ \ \ \ \ \ \ \ \ \ \ \ \ \ \ \ \ \ \ \ \ \ {\rm{\;for\;}}x \le 0.2 \end{array} \right. \end{eqnarray*}$$

${p_{dsDNA}}( {{R_D}} )$ gives the distribution of the dsDNA end-to-end distance (EED) ${R_D}$of a worm-like chain whose contour length is ${L_c}$. In this formula, $N$ is a normalization factor.

The angle distribution of the nDFS-linker is then calculated as follows:

(4) $$\begin{eqnarray*} P\left( \theta \right) &=& {p_{nDFS}}\left( \theta \right) \times \int \left[p\left( {{R_{p1}}} \right)R_{p1}^2\sin {\theta _1}d{R_1}d{\phi _1}d{\theta _1}\right. \nonumber \\ &&\left. \times\, p\left( {{R_{p2}}} \right)R_{p2}^2\sin {\theta _2}d{R_2}d{\phi _2}d{\theta _2} \times {p_{dsDNA}}\left( {{R_D}} \right)\right] \end{eqnarray*}$$

where the three EEDs ${R_{p1}}$,${R_{p2}}$,${R_D}$, nDFS arm length $L$, and nDFS angle $\theta$ satisfy the geometric relationship $2L\sin (\frac{\theta }{2})\; = | {{{\boldsymbol R}_{\boldsymbol {p1}}} + {{\boldsymbol R}_{\boldsymbol{p2}}} + {{\boldsymbol R}_{\boldsymbol D}}} |\;$ and ${p_{nDFS}}( \theta )$ is the free nDFS angle distribution. To numerically calculate the angular distribution of the nDFS-linker, the equation above is discretized. In particular, values of ${R_{p1}}$ and ${R_{p2}}$ are sampled on a spherical grid with spacing $\Delta R\; = \;2$nm, $\Delta \theta \; = \;\Delta R/R$ and $\Delta \phi \; = \;\Delta R/R\sin (\theta )$. The limits are as follows: $0 < R < nb$,$0 < \theta < \pi$ and $0 < \phi < 2\pi$.

We calculated the free energy $G$ from this angle distribution assuming a Boltzmann distribution and obtain the force applied on the incorporated sample by taking the derivative of the free energy with respect to the end-to-end distance, *r*, $F( \theta )\; = \; - \frac{{\partial G( \theta )}}{{\partial r}}$ (details in Supplemental Information Section 1).

This model assumes that the polymer remains in a relatively straight conformation. Since we are using the model to describe the bending of the DNA, we confirmed that the model is valid over our end-to-end distance range of interest by comparing to coarse-grained molecular dynamics simulations performed using oxDNA (36) (Supplementary information, Figure S7). Furthermore, we assumed the Gaussian polymers can be sampled on a spherical grid, but the hinge arms may have some effect on the polymer configuration. We verified that the assumption of the sampling space for the Gaussian polymers has negligible effects on the model outcomes (Supplementary information, Figure S8).

### Prediction of nucleosome unwrapping in nucleosome-nDFS system

Theoretical unwrapping distributions of nucleosomes incorporated in nDFS devices were computed using the experimental angle distribution of the respective empty nDFS in combination with unwrapping free energies calculated via Zhao *et al.* (37). In particular, experimental nDFS angle measurements are sorted into 5^o^ bins to determine the probability associated with each bin ${P_\theta }$. The nucleosome is then described by the numbers *n*_1_ and *n*_2_ of bases unwrapped from each end, respectively, with the angle bin $\theta ( {{n_1},{n_2}} )$ associated with each unwrapping state calculated from the geometry of the model under the assumption that the unwrapped DNA forms a line tangent to the histone at the last point of contact. The total number of unwrapped bases is then divided into 3 bp bins *i*, with the probability density in each bin given by

(5) $$\begin{equation*}{p_i} = \frac{{\sum {P_{\theta \left( {{n_1},{n_2}} \right)}}{e^{ - \frac{{\varepsilon \left( {{n_1},{n_2}} \right)}}{{{k_B}T}}}}}}{{wZ}}\;\end{equation*}$$

where the sum in the numerator is over all combinations of *n_1_* and *n_2_* such that the total ${n_1} + {n_2}$ is within bin $i$, ${P_{\theta ( {{n_1},\;{n_2}} )}}$ is the nDFS probability for the angular bin $\theta$ associated with unwrapping state (*n_1_,n_2_*), $\varepsilon ( {{n_1},{n_2}} )$ is the nucleosome free energy change associated with unwrapping state (*n_1_,n_2_*), $w\; = \;3$ bp is the unwrapped base pair bin width, $Z = {\sum\nolimits_i} {\sum\nolimits_{n{_1}+n_{2}\epsilon i}} {P_{\theta \left( {{n_1}+{n_2}} \right)}} e^{-\varepsilon({n_1},{n_2})/k_{B}T}$ is the partition function associated with the unwrapping states $i$.

### Probing nucleosome unwrapping with the nDFS

To prepare nucleosome samples, recombinant histones were reconstituted with 249 bp of DNA and purified as previously described (22). A modified Widom 601 positioning sequence (Supplementary Table S5) is centered on the DNA, leaving 51 bp of unoccupied linker DNA connecting each side of the nucleosome to the nDFS. This connection between nucleosome and nDFS was made via a biotin-neutravidin linkage. The 5′ ends of the nucleosome DNA were labeled with biotin via primer modification. After reconstitution and sucrose gradient purification, the nucleosomes were bound with neutravidin in a solution containing 10-fold molar excess (to biotin molecules) neutravidin and incubated at room temperature for 15 minutes. The sample was then purified again via sucrose gradient as previously described (22) to remove excess neutravidin before incorporation into the nDFS.

The nucleosome incorporated into nDFS was tested in a buffer containing 50 mM Tris, 200 mM NaCl, and 1 mM MgCl_2_ to mimic physiological conditions. After the PEG-based centrifugal purification nDFS were resuspended to 10 nM in a buffer containing 50 mM Tris, 200 mM NaCl, 2.7 mM MgCl_2_, pH 8.0. Nucleosome samples were diluted to 20 nM in 0.4× TE, pH 8.0, after sucrose gradient purification. Then, 8 μl of the 10 nM nDFS was combined with the 4 μl of 20 nM nucleosomes and an additional 8 μl of a 73 mM Tris and 300 mM NaCl to set buffer conditions. This gave an equimolar final concentration of 4 nM nDFS and 4 nM nucleosomes in 50 mM Tris, 200 mM NaCl and 1mM MgCl_2_. The final solution was incubated on ice for 30 min and then immediately deposited on the TEM grid using the same protocol mentioned above.

## RESULTS

### nDFS design

**Figure 1.**
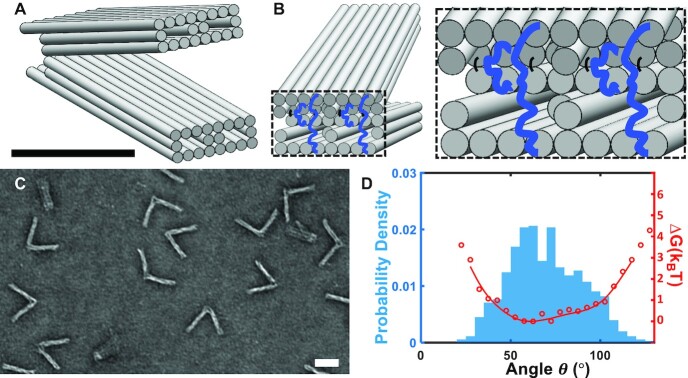
Fabrication and Characterization of the nDFS. (**A**) Isometric view of the nDFS, comprised of two 20-helix bundles with an 8 × 3 square lattice cross-section with 4 helices omitted from the middle layer. (**B**) Isometric view of the back connections of the nDFS. Four connections (black) are 2 nt long, all connecting across the inner layer, and another four (blue) are 70nt long, with two connecting across the inner layer and two connecting across the outer layer. (**C**) Typical TEM image of nDFS version A (nDFS.A) without any additional staples added to the 70nt ssDNA scaffold loops (**D**) Probability density (blue) of the angular conformations of nDFS.A and the corresponding free energy landscape (red) (sample size, *N* = 707, Scale bars 50 nm, Free Energy tick marks denote steps of 1 k_B_T, the minimum of the free energy curve is normalized to zero).

The design of the nDFS was guided by multiple functional requirements, which included the ability to: (i) incorporate one or more biomolecules, (ii) exhibit a wide range of angular distributions to accommodate molecular complexes of different size, (iii) control its mechanical properties via simple design modifications to tune passive force application and (iv) reconfigure the device for active and reversible application of forces. The basic design is comprised of two arms that are ∼61 nm long (from vertex to ends of arms) and made up of 20-helix bundles each organized in 8 × 3 square lattice cross-sections (38,26) with 4 internal helices removed from the middle layer (Figure 1A). The cross-section was inspired by another DNA origami nanostructure that we previously found to exhibit fast and high yield folding (39). The arms are connected at one end by eight ssDNA linkers to form a flexible joint connection. Four of these ssDNA connections are short, 2 nt, and are arranged along a line to form an axis of rotation (black lines in Figure 1B).

A key part of the design is the other four ssDNA connections, which are 70 nt long. Prior studies have shown that the design of the longer ssDNA connections can modulate hinge stiffness (30). In previous hinge designs (40), these long ssDNA linkers connected across the inner layer of helices of the top and bottom arms. In contrast, here we designed the hinge so two of the long linkers connect across the inner layer, and two span across the outer layer of helices (blue lines in Figure 1B). In addition, the arms contain a protrusion of ∼8 nm on the back end. This protrusion prevents excessive opening of the hinge to angles approaching 180°, and it also offsets the two linkers that span across the outer layers from the hinge vertex (Supplementary Figure S9). We reasoned that this design would allow for robust modulation of the hinge properties by changing the design of the 70 nt linkers (e.g., making them partially double-stranded).

In our baseline design, the nDFS.A, the 70 nt linkers are fully single stranded. The nDFS.A device was folded and purified both by gel electrophoresis and by PEG centrifugation (29). Both gel-purified and centrifugation-purified devices were analyzed via TEM. This analysis revealed a notable difference in the angle distributions between the two purification methods. A more detailed study comparing gel-purified (with and without EtBr) and centrifugation-purified devices (with and without EtBr) revealed that the presence of EtBr rather than the purification method per se was responsible for the observed differences in the device properties (Supplementary Figures S10–12). Hence, we focused on centrifugation-purification for the remainder of experiments, including all results presented in the main figures.

Figure 1C shows a representative TEM image of the nDFS.A device. The nDFS angular conformation distribution was quantified from TEM images and plotted as a histogram (Figure 1D). The corresponding free energy landscape was then calculated assuming a Boltzmann distribution. The free energy data was fit to a smoothed spline to approximate the overall free energy landscape. The spline fit was cut off at the edges where we observed relatively few conformations (probability density < 0.001). The probability distribution and free energy landscape reveal flexible angular motion across the range of ∼50–100° with conformations observed with reduced probability down to 25° and up to 125°.

### Modifying scaffold connections enables engineering of free energy landscapes

**Figure 2.**
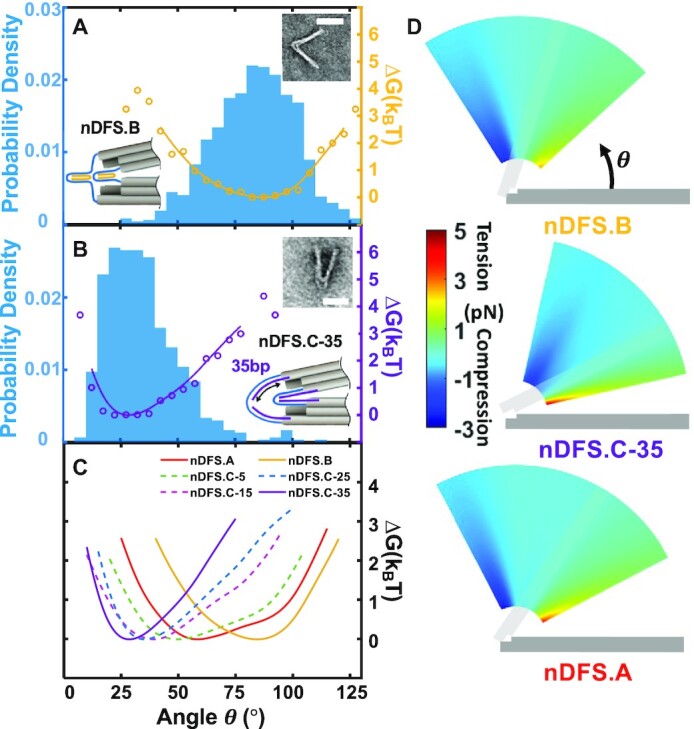
Engineering the nDFS free energy landscape with scaffold connection design. (**A**) Angular probability density (blue) and corresponding free energy landscape (gold) of nDFS.B. Insets show the schematic design of nDFS.B and a typical TEM image. Both the internal and external scaffold loops are pinched by a single staple per loop. (**B**) Angular probability density (blue) and corresponding free energy landscape (purple) for the nDFS.C-35 design shown schematically and in a representative TEM image in the insets. (**C**) Free energy landscapes of the nDFS.A, nDFS.B, and varying nDFS.C designs. (**D**) The force polar contour plot for nDFS.A, nDFS.B and nDFS.C-35. The minimum radial length corresponds to the 32bp typical repeating unit of distance between cross-overs for the DNA origami square lattice structure (∼11 nm). (Minimum sample size, *N* = 498. Scale bars 50 nm, the minima of all the free energy curves are normalized to zero).

We used two general strategies in modifying the 70 nt linkers to tune the nDFS free energy landscapes that relied on adding or substituting a few DNA strand components to either shift towards more open or more closed states. First, to shift towards more open conformations (nDFS.B) we introduced four 60 nt long staple strands that bind to two separate regions of the 70 nt scaffold linkers to pinch the connections into closed loops thereby reducing their end-to-end distance (Figure 2A inset). The nDFS.B exhibited a narrower distribution with most angles in the range of ∼70–110° (Figure 2A), with a mean angle of 83°, which is 13° larger than the mean of the nDFS.A. A direct comparison of the free energy landscapes (Figure 2C, gold and red) of the nDFS.A and nDFS.B reveal a narrow distribution that is shifted significantly to larger angles.

In order to shift the nDFS free energy landscape to more closed states, we followed a strategy based on a study by Sharma et al. (40), who used coarse-grained molecular dynamics simulations using the oxDNA model (36) to show that making the scaffold loops partially double-stranded could bias a hinge device to more closed angles. Rather than introducing staples exclusively on the scaffold linkers (30), we took a distinct approach to extend staple strands directly from the ends of the arms out onto the scaffold linkers (Figure 2B, inset), which we reasoned would improve incorporation efficiency, especially for shorter base-paired lengths on the linkers (minimum length tested was 5 bp). For this nDFS.C design we substituted 8 staples from the nDFS.A device, which bind only inside the hinge arms, with 8 longer staples that extend onto the linkers (Figure 2B, inset). Base-pairing the entire 70 nt linker led to a strong shift to closed angles primarily in the range of 10–50° (Figure 2B). We further tested a range of dsDNA base-pairing lengths on the linkers ranging from 5 through 35 (Supplementary Figure S13). Interestingly, the angular distributions and free energy landscapes revealed that decreasing the length of base-paired DNA on the linker causes a gradual shift back to the nDFS.A behavior (Figure 2C, note that the nDFS.A is effectively the nDFS.C-0). This gradual shift with changes in base-pairing length is likely due to steric interaction of the duplex regions on the linker that have a stronger effect on the free energy landscape for longer duplex lengths.

With the free energy landscape, it is possible to estimate the range of forces that the various nDFS devices can apply. We first calculated the torque as a function of angle by differentiating the free energy landscapes. We limited these torque calculations to the same range as the free energy curves where the angular conformations were reasonably sampled (probability density larger than 0.001, Supplementary Figure S14). Based on these torques, we estimated the forces that the devices could generate on a sample at a particular angle and radial position along the arms (Figure 2D). These calculations revealed a range of forces from ∼3 pN in compression to ∼5 pN in tension (Supplementary Table S6). These forces only describe the range of angles that is sampled in the free device conformational distribution. It is likely that the devices could apply even higher forces at more extreme angles in compression (extreme open angles) or tension (extreme closed angles).

These results demonstrate simple strategies to tune the free energy landscape over a large range of angles from largely open to mostly closed. In particular, the nDFS.C-35 and nDFS.B represent the two extremes, which are almost completely separated in angle range: nDFS.B exhibits 90% of conformations above 60° while the nDFS.C-35 exhibits 93% of conformations below 60°. Importantly these large changes in properties can be achieved by substitution of just a few of the strand components to modify the design of the scaffold linkers at the hinge vertex. This versatile modulation of the nDFS free energy landscape provides a basis for tuning forces applied to incorporated molecular complexes or particles that constrain the state of the device (e.g. latch the arms into a relatively closed state (22,41)).

### Internal dsDNA struts constrain the angle of the nDFS

In addition to engineering the mechanical properties, we also developed an approach to actuate reconfiguration of the nDFS as a basis for active application of forces. We chose to demonstrate the actuation with the nDFS.B device since its angle distribution is strongly biased to large angles, hence it is easier to detect a conformational change between open and closed states.

**Figure 3.**
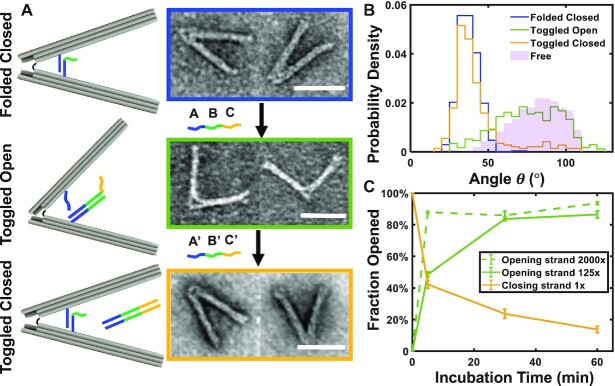
Reversible toggling between open and closed states of the nDFS.B. (**A**) Schematics and representative TEM images of the nDFS.B at various stages of toggling. Top (Folded Closed): nDFS.B structures were folded with internal struts, comprising a 19 bp duplex, to restrict the range of angular motion. The bottom strut overhang has an additional 10 nt that remain single-stranded (green domain labeled B’) to serve as a toehold for the opening actuation. Middle (Toggled Open): The nDFS is toggled open by introducing opening strands, marked as ***ABC***, which are complementary to the 10 nt toehold (***B***-domain) and to 16 nt of the bottom strut overhang (***A***-domain). The opening strand also contains an additional 10 nt that remained single stranded (***C***-domain) to serve as a toehold for the closing actuation. Bottom (Toggled Closed): the nDFS is toggled closed by introducing closing strands, marked as ***A′B′C′***. The closing strand is fully complementary to the opening strand. Right: Typical TEM images of each state. (**B**) Histograms comparing the angular probability distributions of different states. Free nDFS (nDFS.B without strut, light purple) is also shown for reference. (**C**) Timescale measurement of nDFS toggling open or closed by varying strand concentrations. x represents the excess strand concentration with respect to nDFS. (Minimum sample size, N = 292, Scale bars = 50 nm).

The nDFS.B device was modified by adding a set of struts, which consist of a 19 bp dsDNA duplex that forms by base-pairing between two DNA overhangs, one that extends out of the top arm and one that extends out from the bottom arm. We incorporated two struts in parallel, both ∼12 nm away from the hinge vertex (Supplementary Figure S15). This was chosen to achieve a closed state angle of ∼30°, to make it easy to distinguish from the ∼83° open state of the free nDFS.B device. The bottom strut overhangs have an additional 10 nt that are not complementary to the top strut overhang and remain single-stranded to serve as a toehold (green line in Figure 3A) for subsequent DNA strand displacement (42) to toggle into the open configuration. Prior work has shown the overall rate for strand displacement saturates at toehold lengths of 6–8 nt (43). However, given that the strut overhangs are coupled between two arms of the nDFS, the toehold length was deliberately designed longer to increase accessibility.

We first tested directly folding into the closed state, which we refer to as Folded Closed (Figure 3A, left). As illustrated in the angle distribution (Figure 3B, blue line), the Folded Closed nDFS.B devices exhibited conformations shifted to smaller angles relative to the free nDFS.B, suggesting effective incorporation of the strut with high efficiency (Figure 3B, Supplementary Figure S16). To actuate the nDFS.B to the Toggled Open state, opening strands, which are complementary to both the B’ toehold and the A’ domain of the bottom arm overhang, were introduced (125-fold molar excess relative to nDFS). We estimated a toggling open efficiency of 82% by measuring how many devices exhibited an angle that is larger than the maximum of the Folded Closed angular distribution (i.e. angles larger than 55°). This efficiency is a lower bound since some free nDFS.B devices may still exhibit angles lower than 55°. The opening strands also contained an additional 10 nt that remained single-stranded to serve as a new toehold (orange C-domain on opening strand in Figure 3A) for the second step of DNA strand displacement.

To toggle back to a closed state, the closing strands, which are fully complementary to the opening strands, were introduced (equimolar relative to nDFS). Once the opening strands are removed, the strut overhangs can re-bind to restrict the range of angular motion. We refer to this closed state as Toggled Closed (Figure 3A). Note that the closing strand can also bind to the top arm strut overhang, which could inhibit re-formation of the internal strut. To circumvent this potential issue, the A and A’ domains on the Opening Strands and Closing Strands were designed to be only 16 nt long, so direct binding of the 19 nt strut overhangs could outcompete the binding between the closing strand and the top arm overhang (Supplementary Figure S17). Equimolar concentrations of the closing strands relative to the nDFS.B yielded a toggling efficiency of 87% (Figure 3B), which we estimated by measuring how many devices exhibited an angle that is smaller than the maximum of the Folded Closed angular distribution (i.e. angles less than 55°). Increasing the closing strands concentration did not increase the toggling efficiency (Supplementary Figure S18), likely due to binding between the closing strands and the top arm strut overhang. These results suggest that the nDFS can be toggled back and forth between free, or open, and closed states at high efficiency (>80%), providing a basis for triggered (i.e., active) application of forces.

In addition, we performed experiments to measure the timescale of toggling by performing TEM imaging characterization at several time points (5, 30, 60 min). For simplicity, the toggling open (i.e. Folded Closed to Toggled Open, green curve in Figure 3C) and toggling closed (i.e. Folded Open to Toggled Closed, orange curve in Figure 3C) processes were tested separately. For the toggling open, we tested two excess ratios of opening strand, 125-fold and 2000-fold excess relative to nDFS (nDFS at 1 nM). We found that the opening actuation can reach 88% efficiency within 5 min with the 2000-fold excess of opening strand, while the125-fold excess took 30 min to reach a high opening efficiency of 84%. For the closing actuation we only tested the equimolar concentration, since increasing the concentration of closing strand did not speed up the actuation (Supplementary Figure S18). The closing actuation reached 58% efficiency within 5 min and 76% efficiency with 30 min.

### The nDFS can exert compressive forces up to pN scale on dsDNA molecules

Applying compressive forces with traditional single molecule methods is challenging because of the large mismatch in size between the force probe (e.g., bead or cantilever tip) and sample and the resulting need for long compliant handles, often flexible dsDNA tethers, to attach the molecular sample to the probe and/or surface. Hence, we tested the capability to apply compressive forces to demonstrate this unique capability of the nDFS. To allow for incorporation of a test sample, the nDFS.B was modified with a biotin molecule at the end of each arm. We used a 249 bp (or 85 nm) dsDNA molecule as a test sample. The persistence length of dsDNA is ∼50 nm (32–34), which suggests it should remain relatively straight in the absence of an applied load. Indeed, a direct measurement of free dsDNA samples from TEM images revealed an end-to-end distance (EED) of 62 ± 11 nm (mean ± standard deviation, Supplementary Figures S7B and S19D). Hence, this dsDNA sample is similar in length to the distance between the free ends of the hinge arms of the nDFS.B (82 nm for the peak angle of 85°), which likely allows for efficient incorporation.

**Figure 4.**
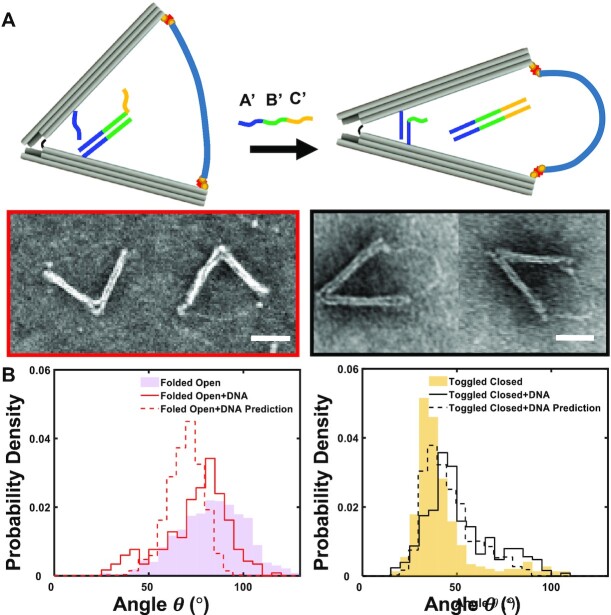
Applying compressive forces to dsDNA. (**A**) Schematic illustrating the application of a compressive force on a dsDNA sample incorporated into the nDFS.B. The nDFS.B devices were folded directly into an open state (Folded Open). A 249 bp dsDNA is incorporated by binding across the ends of the arms. Then, the closing strands are introduced, thus allowing the two complementary internal strands to bind to each other actuating the hinge into the Toggled Closed state and deforming the dsDNA sample (scale bars = 50 nm). (**B**) Histograms comparing the angle distributions for open and closed nDFS both in the presence and in absence of dsDNA. Solid lines show experimental measurements, while dashed lines show theoretical predictions based on a WLC model of the dsDNA sample (minimum sample sizes, *N* = 234).

The nDFS device was directly folded into the open state (Folded Open) with biotin molecules included at the ends of the arms. The dsDNA sample, which contained biotin at both ends, was functionalized with neutravidin and purified using a sucrose gradient. Then, it was incorporated via biotin-neutravidin binding by incubating the dsDNA sample for 30 min at 37°C leading to an incorporation efficiency of 64% as quantified by inspection of TEM images (Figure 4A and Supplementary Figure S5). Binding of the dsDNA sample led to a slight shift to smaller angles (Figure 4B left, solid red line), which is expected since the EED of the free dsDNA sample is slightly shorter than the distance between the ends of the arms for the peak of the free nDFS.B distribution. We then toggled the nDFS.B into the closed state using the same protocol as previously described. Toggling the nDFS closed induced a large bend in the dsDNA sample (Figure 4A), similar to a post-buckling shape of a slender beam (44). The angle distribution of the toggled closed sample with the dsDNA (Figure 4B right, solid black line) is slightly shifted (∼6° at the peak) to larger angles relative to the toggled closed sample without dsDNA, because the dsDNA resists bending and applies a force acting to open the hinge.

To better quantify the DNA compression, we implemented a theoretical model based on a stiff polymer approximation to the wormlike chain model (35) to predict the behavior of the nDFS with the compressed DNA. The polymer model gives a probability corresponding to each DNA EED, which agrees well with our experimentally measured EEDs. This EED represents the distance between the ends of the hinge arms, and therefore corresponds to a specific hinge angle. The probability distribution of this angle in the absence of DNA is observed empirically, as shown in Figure 3B. In order to predict the angle distribution in the presence of DNA, we combine the probabilities of the DNA EED and the no-DNA hinge distribution at each angle following an approach similar to that of Zhao *et al.* (37). The predicted angle distributions (represented by dashed lines in Figure 4B, free energy landscape in Supplementary Figure S20) agree qualitatively with experiments. For the open state, the model reveals a shift towards smaller angles, but the predicted shift is ∼10° larger than the experimentally observed shift at the peak of the distribution. This discrepancy can likely be explained by fraying of the hinge ends that typically occurs at the ends of DNA origami bundles (45,46), which would allow for a larger angle to accommodate a given dsDNA sample end-to-end distance (Supplementary Figures S21 and 22). In the closed state, the prediction agrees quite well with the experimental distribution, although the peak angle is about 5° larger in the experiment relative to the prediction. While the model and the observed data differ somewhat on the peak position, they agree reasonably well on the tail of the distribution. Since the model assumes that the presence of the dsDNA does not affect the actuation, this indicates that the dsDNA does not significantly interfere with toggling closed, perhaps with the exception of the small increase in devices exhibiting angles around 90°.

Since this worm-like chain approximation reasonably describes the behavior of the DNA compression sample, especially in the closed state of the nDFS, we used the model to estimate the compressive force exerted on the DNA by the nDFS. To estimate the force, we determined the free energy ($G$) versus end-to-end distance ($r$) of the DNA sample, and calculated the force as $F\; = \; - dG/dr$ evaluated at the appropriate end-to-end distance of the arm tip corresponding to the bent configuration in the nDFS experiment. This estimate of the force required to deform the DNA into the highly bent state corresponding to the peak of the distribution (solid black line in Figure 4B, right) yields 0.28 ± 0.06 pN.

We also observed that the conformation of the dsDNA sample in the closed nDFS state resembled a largely bent post-buckling beam conformation. Hence, we also estimated the force on the dsDNA sample using an Euler Elastica model (44) that describes large bending deformations of slender beams (additional details provided in Supplemental Information, Figures S23, S24,Table S7). This approach yielded a compressive force of 0.4 ± 0.1 pN, which agrees well with the force estimated from the wormlike chain model.

### The nDFS can exert tensile forces to induce varying amounts of unwrapping in nucleosomes

**Figure 5.**
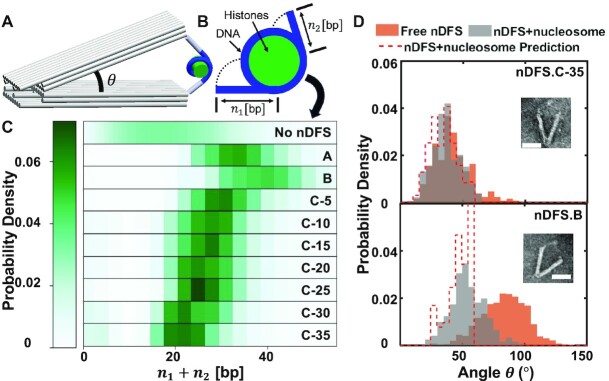
Nucleosome unwrapping induced upon incorporation into various versions of the nDFS. (**A**) A three-dimensional model of the nucleosome-nDFS system. (**B**) Schematic of the free nucleosome illustrating the number of unwrapped base pairs on each side, given by *n*_1_ and *n*_2_. (**C**) The prediction of total unwrapped base pair probability density for all the nDFS devices tested, as well as a free nucleosome. (Nucleosome unwrapping in the absence of nDFS is due to thermal fluctuations). (**D**) The angular distributions for nDFS.C-35 and nDFS.B comparing between free nDFS, nDFS in the presence of nucleosome, and the model prediction.

In addition to active application of forces via toggling, the ability to tune the free energy landscape of the nDFS also provides a platform for physical studies of biomolecules. For example, for a molecular complex incorporated between the arms of the device, the nDFS.B device could bias the complex toward larger end-to-end distances, while the nDFS.A or nDFS.C-5 could sample a broad range of end-to-end distances, and the nDFS.C-35 could bias the complex toward shorter end-to-end distances. To illustrate this utility, we predicted and validated how nDFS devices could modulate the unwrapping conformations of nucleosomes, building on prior studies using DNA origami devices to study nucleosome unwrapping (22,47). We used the force spectrometer free energy landscapes determined in this study in combination with a previously established nucleosome model that describes the unwrapping of nucleosomal DNA in the context of a DNA origami hinge (37) (description of model provided in Materials and Methods) to predict how a range of nDFS devices could modulate nucleosome unwrapping conformations. We found the nDFS devices could induce various degrees of unwrapping from a total of ∼20 bp (nDFS.C-35) to a total of ∼40 bp (nDFS.B) (Figure 5C). The small degree of unwrapping observed for the free nucleosome is due to thermal fluctuations, since the free energy associated with initial unwrapping is on the scale of thermal energy (48).

We selected the two extremes, nDFS.B and nDFS.C-35, for experimental validation (Figure 5D, Supplementary Figure S25). Nucleosomes were incorporated into nDFS devices and angular distributions characterized as previously described (22) (details in Materials and Methods). The experimental angle distributions agreed well with the model predictions, especially for the nDFS.C-35 where the model accurately predicted the full distribution (Figure 5D, top). For nDFS.B, the model accurately predicted the peak angle and the main experimental population, but we observed a small population of larger angles in experiments, which was not captured by the model. This is likely due to histone H2A-H2B heterodimer dissociation, which has previously been shown to be facilitated by applied forces (49,50).

To estimate the tensile forces applied to these nucleosome samples, we differentiated the free nDFS energy landscape at the location corresponding to the most likely conformation when the nucleosome is incorporated in the nDFS device (e.g. in nDFS.B, at an angle of 53° for the gray data in Figure 5D). The error bar was determined by bootstrapping nDFS and nucleosome data (gray data). The tensile forces were estimated for nDFS.B and nDFS.C35 as 0.37 ± 0.02 pN and 0.16 ± 0.02 pN, respectively. Since the force exerted by nDFS would be the same as the force experienced by the nucleosome, alternatively, we also estimated the tensile forces by differentiating the free nucleosome free energy landscape. The number of unwrapped base pairs for the free nucleosome due to thermal fluctuations was converted into an angle by assuming the nucleosome was incorporated into the nDFS with a flat energy landscape (i.e. no force applied at any angle) (37). This yielded tensile force estimates for nDFS.B and nDFS.C-35 as 0.34 ± 0.03 and 0.14 ± 0.01 pN, respectively.

These results are consistent with prior studies that show forces on the scale of ∼1 pN are sufficient to cause initial unwrapping (51). Furthermore, these experiments illustrate how a set of nDFS devices can be used to probe various regions of the conformational distribution of a sample molecule without the need for external actuation.

## DISCUSSION

Scaffolded DNA origami allows for the development of nanoscale devices with Angstrom resolution that have been increasingly used for biophysical measurement applications (17–19). However, prior devices have limitations such as the inability to tune forces or actively apply forces (i.e. no actuation), and they still are limited to tension. Here, we expand on these studies by adjusting mechanical properties (i.e. average angle and hinge stiffness) of a caliper device over a large range by substituting just a few strand components at the hinge vertex. We further developed an active approach to apply forces that relies on triggered reconfiguration of the DNA device based on a removable strut system. In this study, actuation occurred on the timescale of several to tens of minutes (Figure 3C). Hence, devices could be used to study processes that occur at similar or slower timescales under varying mechanical loads. We designed the strut with mechanical integrity in mind using multiple duplex interactions and avoiding nicks in the strut by using overhangs that directly bind to each other. It is likely the strut design and experimental conditions could be optimized to further speed up actuation (52), but design changes that improve actuation response time may lead to a more flexible strut. In addition, the device could be used to study faster processes under constant load. In these cases, the advantage of the toggling is the ability to incorporate a sample under conditions that allow efficient binding (e.g. open angle for the large dsDNA sample) and then apply mechanical loads over an extended amount of time.

We developed two approaches for force application. First, we demonstrated active application of compressive forces to bend a dsDNA sample, which required ∼0.1–1 pN of force. This force was sufficient to induce a large deformation of ∼36 nm to cause a highly bent state of dsDNA, which is biologically relevant since many proteins induce bending (53) or preferentially interact with bent DNA (54). We also demonstrated application of tensile forces to modulate nucleosome unwrapping configurations, which was achieved by incorporating nucleosomes that restricted nDFS devices to smaller angles than the free device. These tensile forces were also on the scale of ∼0.1–1 pN and were sufficient to induce up to ∼40 bp of unwrapping (Figure 5). The resulting forces applied by the nDFS onto the sample depend on the device properties and the sample size, compliance, and location of incorporation, which all influence the resulting hinge conformation. The two cases we demonstrated utilized compliant samples that could undergo large deformations (tens of nanometers) with just ∼1 pN of applied force. Stiffer samples that cause the hinge to adopt higher free energy configurations would lead to higher forces. Based on our free energy analysis, we estimated the hinges could apply forces up to ∼3–5 pN over the range of angles that are sampled in our angular distributions which is comparable to forces induced by molecular machines (16). It is likely that the nDFS is capable of applying even higher forces at more extreme angles, although our results suggest accounting for local deformations (e.g. fraying) would likely be important at higher forces when samples are incorporated at the ends of the arms.

Our study also revealed that EtBr, which is widely used during DNA origami purification, can influence the properties of flexible structures, which is an important result given the increasing interest in dynamic DNA devices (55). This is likely due to the influence of EtBr on length and stiffness of DNA (56,57). In particular, given the strong dependence of the nDFS properties on the details of the hinge vertex design, changes in properties of the scaffold linkers due to EtBr interactions could cause changes in device properties. Centrifugal purification in the presence of PEG (29) provides an effective alternative to avoid this effect with the added benefit of being able to control the structure concentration after purification. Gel purification without EtBr is also suitable alternative, especially for cases where removing misfolded or aggregated structures is necessary.

Given the large body of prior work integrating biomolecules into DNA origami devices (22), the nDFS could be used for a wide range of biophysical studies including the unique capability of applying compression. The experiments carried out in this study were performed at relatively high ion concentrations compared to physiological conditions. However, gel analysis revealed the nDFS is stable for more than several hours in both physiological salt conditions (1mM MgCl_2_ and 200 mM NaCl) and cell culture media even with high levels of serum (RPMI supplemented with 50% FBS) (Supplementary Figures S26 and 27), suggesting the devices are compatible with biologically relevant conditions. Furthermore, since the force is applied directly with the nanoscale device, the nDFS can synergistically work with other biophysical methods such as single-molecule fluorescence to probe the dynamics of interactions or even other force spectroscopy methods for example to amplify the range of forces or control the direction of loading. In addition, the ability to directly apply tunable forces with a nanodevice could be leveraged in combination with a variety of imaging techniques such as super-resolution fluorescence microscopy or cryogenic transmission electron microscopy to study deformed molecules at high resolution. Here we utilized negative stain transmission electron microscopy to image the nDFS angle distributions, which is an instrument often available at user facilities. Furthermore, AFM has also been used to quantify DNA origami conformations; and moving forward other methods, such as Förster resonance energy transfer (FRET), could be used to quantify nDFS states. This provides multiple readout options, which can significantly broaden access to force spectroscopy studies and likely lower cost when instruments are available locally or in user facilities. Finally, given the size and biocompatibility, in the future these devices could even be implemented for mechanical studies of biomolecules in their native cellular environment.

## DATA AVAILABILITY

The experimental data sets are either included in this submission, the supplemental information, or are available from the authors upon request.

## Supplementary Material

gkab656_Supplemental_File
