# Supplementary material for: A nanoscale DNA force spectrometer capable of applying tension and compression on biomolecules
Source: Nucleic Acids Res. 2021 Aug 6;49(15):8987–99. doi: 10.1093/nar/gkab656 (PMC8421221; doi:10.1093/nar/gkab656)
Supplement: gkab656_Supplemental_File [file gkab656_supplemental_file.pdf]

Supplementary Material

A Nanoscale DNA Force Spectrometer Capable of Applying Tension and Compression on Biomolecules

Yuchen Wang[1†], Jenny V. Le[2†], Kyle Crocker[3], Michael A. Darcy[3], Patrick D. Halley[1], Dengke Zhao[3], Nick Andrioff[4], Cassie Croy[1], Michael G. Poirier[2,3,6], Ralf Bundschuh[2,3,6,7], Carlos E. Castro[1,2,*]

[1] Department of Mechanical and Aerospace Engineering, The Ohio State University, Columbus, Ohio 43210, United States
[2] Biophysics Graduate Program, The Ohio State University, Columbus, Ohio 43210, United States
[3] Department of Physics, The Ohio State University, Columbus, Ohio 43210, United States
[4] Department of Chemical and Biomolecular Engineering, The Ohio State University, Columbus, Ohio 43210, United States
[6] Department of Chemistry and Biochemistry, The Ohio State University, Columbus, Ohio 43210, United States
7 Division of Hematology, Department of Internal Medicine, The Ohio State University, Columbus, Ohio 43210, United States

Supplementary Material Table of Contents

Figure S1 caDNAno schematics of nDFS. 4

Table S1 nDFS Main Structure Staples 4

Table S2 nDFS Staples that bind on scaffold linkers in various design versions. 10

Table S3 Other nDFS staples 13

Figure S2 Agarose gel showing nDFS.A folded in [12,14,16,18,20,22,24,26] mM MgCl2 with 1kb ladder and p8064 scaffold as a control. 18 mM $MgCl_2$ was chosen for all subsequent folding reactions. 14

Figure S3 Representative TEM images of (A) nDFS.C-5, (B) nDFS.C-10, (C) nDFS.C-15, (D) nDFS.C-20, (E) nDFS.C-25, (F) nDFS.C-30, (G) nDFS.C-34, (H) nDFS.C-35, (I) nDFS.B. (Scale bars = 100 nm). 15

Figure S4 Sample TEM image galleries of (A) nDFS.C-5, (B) nDFS.C-10, (C) nDFS.C-15, (D) nDFS.C-20, (E) nDFS.C-25, (F) nDFS.C-30, (G) nDFS.C-34, (H) nDFS.C-35, (I) nDFS.B. (Scale bars = 60 nm). 16

Table S4 Sample Sizes in TEM Characterization 17

Table S5 249 bp dsDNA sequence 17

Figure S5 dsDNA binding efficiency to nDFS in both closed and open states quantified by TEM. 18

Section 1: Additional details on DNA Compression Prediction Model 19

Figure S6 nDFS-dsDNA schematic. 19

Figure S7 (A) oxDNA correlation test. 20

Figure S8 Local spherical coordinate system schematic. 21

Figure S9 The back view of nDFS bundle model showing details of scaffold routing. 22

Figure S10. nDFS angular distribution results are influenced by purification method. 23

Figure S11 EtBr effect on nDFS.C-20. 24

Figure S12 EtBr effect on nDFS.C-25. 25

Figure S13 The angular distributions and free energy landscapes of nDFS.C with different lengths of the staples that extend out onto the scaffold linkers. 26

Table S6 The forces generated by multiple versions of nDFS within limited sampling region. 26

Figure S14 The calculation of torque versus angle for different versions of nDFS based on their free energy landscapes. 27

Figure S15 Internal strut locations. 28

Figure S16 Representative TEM images of nDFS.B.. 29

Figure S17 Toggling the nDFS by actuating the strut. 30

Figure S18 Toggling efficiency with varying displacement staple concentrations. 31

Figure S19 Representative TEM image galleries of structures within the compressive force workflow. 32

Figure S20 Free energy landscape comparison for open and closed nDFS both in the presence and absence of dsDNA. 33

Section 2: Fraying may explain discrepancy between experimental and predicted open hinge distributions 33

Figure S21 Fraying of nDFS. 35

Figure S22 caDNAno design of ends of nDFS arms. 35

Section 3: Solving the Elastica Model for Post-buckling Large Compression 37

Figure S23 Beam deflection under horizontal load P 37

Figure S24 Influence of dsDNA persistence length on compression force 38

Table S7 Basic parameters used in the Elastica model 38

Figure S 25 Representative TEM image galleries of structures with nucleosomes 39

Figure S26 nDFS stability in serum and varying ion conditions. 39

Figure S27 FBS TEM Images. 40

Reference 41

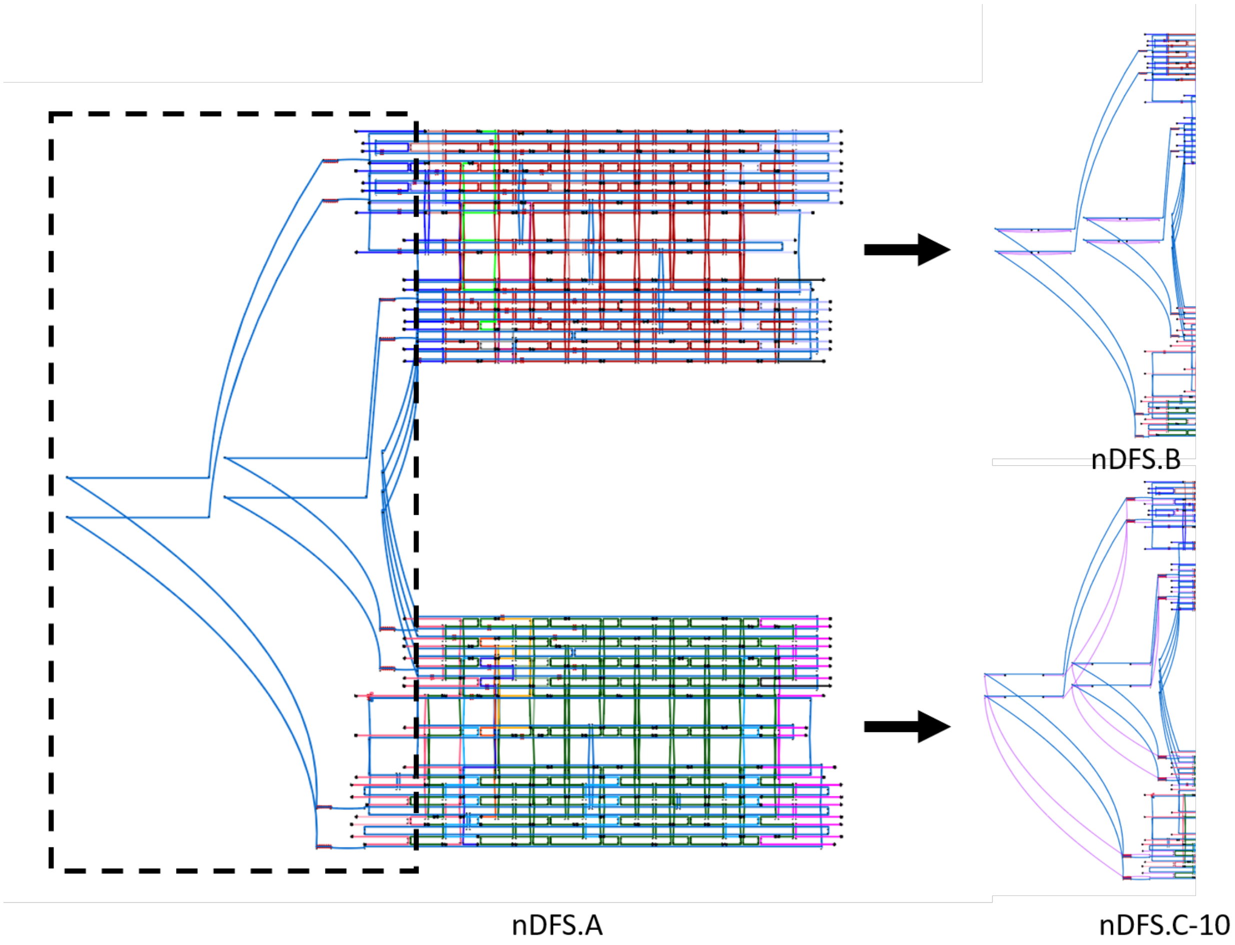

Figure S1 caDNAno schematics of nDFS. The differences among the various versions of the nDFSs are the specific design of the 70 nt linkers at the hinge vertex. In the nDFS.A, the linkers are fully single-stranded. The nDFS.B design contains four staples, each of which binds to one scaffold linker to pinch the linker into a loop. In the nDFS.C designs, staples at the left end of the arms are extended out onto the linker to base-pair a certain length (e.g. in the nDFS.C-10, the staple base-pairs 10nt of the linker).

Table S1 nDFS Main Structure Staples

**Staple Sequence**	**Element**	
**TTTTTT**CAGTTTGGAACAAGAGTCCACTATTAA	**Arm Staple 1**	
TATACTTCGTATGGGATCTAAAGTTTTGTCGTCTTTCCAGAC**TTTTTT**	**Arm Staple 2**	
**TTTTTT**AAGTGTTTTTATAATACGCCAGAATCCTGAG**TTTTTT**	**Arm Staple 3**	
AAGTTTCCGAAGGCACCAACCTAAGCGTCCAATACTGCGGAATCGTCATAAAT ATTCA	**Arm Staple 4**	
**TTTTTT**GTTAGTAAATGAATTTTCTTGAATAATGGAAGGGTTAG	**Arm Staple 5**	
**TTTTTT**TACGTAATGCCACTACATTAAACGGGTAAAA**TTTTTT**	**Arm Staple 6**	
GCCCGAGATAGGGTTGAGTGTTGTTC**TTTTTT**	**Arm Staple 7**	
**TTTTTT**ACAAAGAAACCACCGTAACGATTTTGCTTCATGAGG	**Arm Staple 8**	
**TTTTTT**ATAACGCCAAAAGGAATTACGGACTGGATAAACGAAA	**Arm Staple 9**	
**TTTTTT**ACACAACATACGAGGCTGGAGGTGTC	**Arm Staple 10**	

**TTTTTT**GATAGCTCTCACGATCATTTTGCGGA**TTTTTT**	**Arm Staple 11**	
**TTTTTT**GCCAGAATGGAAATGATAATCAGAAA	**Arm Staple 12**	
**TTTTTT**CCTGAGAGTCTGGTAATGCAGATAC**TTTTTT**	**Arm Staple 13**	
TACCGAGCTACACTGGTGTGTTCAACAATCGGCGAAACTGCT	**Arm Staple 14**	
TTCGCGTCCGTGAGCCCATCAGATGCCGGGTTGAGCCGCC	**Arm Staple 15**	
CCGGGGGTTTCTGCCAGCCGGTGCCCCCTGCAAAACGACG	**Arm Staple 16**	
GCGCCGTTTTCACCTTATCA	**Arm Staple 17**	
TGCGCGCCAACGCCAGGGTTTTCCGCGAAAGGATCGCAAG	**Arm Staple 18**	
CCATGTTTCGTCATAAACATCCCTTCGAATTCCCTGTTTA	**Arm Staple 19**	
TCACCGGAAGCAAATCGTTAACGGTCCTCACAAGAAAAAT	**Arm Staple 20**	
ACGGGAACATTCAGGCCTAAATTTATCAAAATCATGATTAGCCAAAGA	**Arm Staple 21**	
CAGAGGTGACCTGCAGCCAGCGGTGCACGCGTATGTAGAA	**Arm Staple 22**	
GCCAGTGCCGGTGCGGCAAATATATAACCTCCCGCAATAAGAGGGAGG	**Arm Staple 23**	
ACAGGTGAGAGATAGACTTGCGGCTGGTAATGGGTCTGTGTGATAAACAA	**Arm Staple 24**	
TCTGATTACCTGTTAAGACGAGCAAACGCAATCAATAGAA	**Arm Staple 25**	
GCCAGCTGCAGTCACGACGTTGTATCAGACGATCCA	**Arm Staple 26**	
GAAGTACCTTTTTTTTAGTTAACGAATTGAGTTAAG	**Arm Staple 27**	
GTGCATCTCCGTAATGGGATAGGTCATATATT	**Arm Staple 28**	
GGCCTTCCAACCGTTCTAGCTGAGAATTAGAGATACAT	**Arm Staple 29**	
AAATGTGAGGAGACAGTCAAATCAAGCATAAATTCTGCGA	**Arm Staple 30**	
CCGTGGGAGTAGGTAAAGATTCAATTATGACCAAGTTTCA	**Arm Staple 31**	
TACAAAGGTTAAATCAGCTCATTTGATA	**Arm Staple 32**	
ATGATATTCTGTAGCCAGCTTTCACACCGCTTGTGAATTTAATGGTTT	**Arm Staple 33**	
CAAGGCAAATAAATTAATGCCGGACGCCATCAAAAATAATTAATTAATAGCT	**Arm Staple 34**	
CAAAAACAAAGGGTGAGAAAGGCCGCGAGTAACAACCCGTTCAG	**Arm Staple 35**	
GGAGAAGCAATGCCTGAGTAATGTACAAACGGCGGATTGAGCCA	**Arm Staple 36**	
CATGTTCAGGGAGGTTCGAGCGTCTTTCCAGAACGCTCAA	**Arm Staple 37**	
TCAACAATCGCGAGGCACAAAATAAACAGCCAGCGTTATA	**Arm Staple 38**	
AATATCCCAGATATAGCCAAATAAGAAACGATAATTACTA	**Arm Staple 39**	
ACCAATCAGAATCATTAAAATGAAAATAGCAGTATCAGAG	**Arm Staple 40**	
TTCCAAGAGAACAAGCAACATAAAAACAGGGACTGAACAAAGTCAGAG	**Arm Staple 41**	
GAAATTATTCATTTCATAACCAGGCAAAGCGCCATTCGCCGGATAACC	**Arm Staple 42**	
CTTCTGACTGCGCAACCCAGCCAGCTTTCCGGTCAACATT	**Arm Staple 43**	
AACTTTTTGCCTCTTCGACGACAGTATCGGCCCGGATTCT	**Arm Staple 44**	
TAGCGATTTTCCCTTTGTGAGTGAATAACCTGTACAGCG	**Arm Staple 45**	
TAGAAGCAAAAGAAGTTACATACCAGTATAAAGCCAGCCTAATT	**Arm Staple 46**	
AGTCAATACTGGTGCCGGAAAATCGTCGCTATTCGCGTCT	**Arm Staple 47**	
TGAGAGACATCGCACTTGTTGGGAAGGGCGATCAAGCTTT	**Arm Staple 48**	
TATGTAAACGAACAAATTCATTAAAGGTGAATTTAGAGCC	**Arm Staple 49**	
TTCGCAAAGCGTTTTACTTTAGCGTCAGACTGTAAGTTTA	**Arm Staple 50**	
TAGTTTGACCATTCAAAATTAAGCAATAAAGCCTCAGCCATCAAT	**Arm Staple 51**	
ACGAGTAGCCGGAAGCACCGTAAT	**Arm Staple 52**	
TTCCATATGAGTACCTGAAACGTCACCAATGACGACATTC	**Arm Staple 53**	

ATGCAACTTCATTTTTCACCAGTA	**Arm Staple 54**	
TTAAATCAAGATTAGTGTCCAGAC	**Arm Staple 55**	
TCCGGTATTCTAAGAAAGATAAGTCCTGAACAGTTGAGGATCCCCGGG	**Arm Staple 56**	
CAATAGCAAGCAAATCATCCTAATTTACGAGCGCCTGTTC	**Arm Staple 57**	
TTTATTTTCATCGTAGATAATCGGCTGTCTTTCGGTCATA	**Arm Staple 58**	
TATCCTGACATATTTAACAACGCCAGTACCTTTTACATCGCGCC	**Arm Staple 59**	
TGCCAGTTGTTTTAGCGAACCTCCCGACTTGC	**Arm Staple 60**	
CAGTAGGGATTCGCCTGATTGCTTAAAGGTGGTGAAAACA	**Arm Staple 61**	
CAAATTCTAAATCGCGCAGAGGCGAGTATGTTCTGAGAAG	**Arm Staple 62**	
GAAAAAGCTAAATAAGGCGTTAAAAGAACTGGCATAGGTC	**Arm Staple 63**	
AGATAACCCACAAACCGGAATCATTTTTTGTTTAACGTCAACCGCGCC	**Arm Staple 64**	
CCCAATAACGAGGAAAGGCTTAGGTTGGGTTAGAGGGGACGCTATTAC	**Arm Staple 65**	
ACCCGCATTGACAGGAGGTTCAAACAAATTTTTAATGGAA	**Arm Staple 66**	
AAGGAAACTAAGAGCACGCGAGAA	**Arm Staple 67**	
CGCCTCCCCCCCCTTATTAGCGTTAGCAAAGCGGATTGCA	**Arm Staple 68**	
CAAAGACACCACGGAATAGCGCGTTTTCATCGGAAGCCCGAAAGACTT	**Arm Staple 69**	
TTTTGTCATAGAAAATACATACATTGAATACC	**Arm Staple 70**	
AATTACAGAATCAAGTTTGCATTCGAGCTTCAAAGCGAACCAGAATT	**Arm Staple 71**	
CAAAAGGGAACCATCGATAGCAGCAAACTCCAACAGGTCA	**Arm Staple 72**	
AACCGATTTAACGGAATACCCAAATAAGAATAAATTTCAT	**Arm Staple 73**	
GAAGGTAAACCATTAGCAAGGCCGTTAATTGCTCCTTTTG	**Arm Staple 74**	
CAAATATCTGGTCAATAACCTGTTCAATAAATCATACAGG	**Arm Staple 75**	
GGATTAGAAACAGTTGATTCCCAAGCTAAATCGGTTGTAC	**Arm Staple 76**	
ATAAGAGGAAAGTACGGTGTCTGGCTGTAATACTTTTGCG	**Arm Staple 77**	
TTCAGAGGCAGGAAACAAAAAATAACGGCTTAATTG	**Arm Staple 78**	
GTTTTATAACTAACAAAGAAAGAAACAAGGTAATTG	**Arm Staple 79**	
AGAATCGCATCTTACCAACGCTAATTGAAGCC	**Arm Staple 80**	
TATCATATTATTATTTATCCCAATAAGGCTTA	**Arm Staple 81**	
AGCGCTAACCTTTACAGAGAGAATAAGCCGTT	**Arm Staple 82**	
TATTACGCAATACCGACCGTGTGACTGTTTAG	**Arm Staple 83**	
GCATTTTCGGTCATAGTCAGAGCCGCCAAACGAAAAGACC	**Arm Staple 84**	
CAGTAGCGCATATGGTTTACCAGCAGACTCCT	**Arm Staple 85**	
GCACCATTATATTGACGGAAATTAGTTACCAG	**Arm Staple 86**	
AAATCAAGGGCGAAAATCCTGTTTGTGAGCTAACTCACAT	**Arm Staple 87**	
AGCACTAACAGCAAGCGGTCCACGACTGCCCGCTTTCCAG	**Arm Staple 88**	
GATTTAGATTGCCCTTCACCGCCTGCCAGCTGCATTAATG	**Arm Staple 89**	
CGGCGAACGTGGCGAGTTCTTTTCACCAGTGAGGGAGAGG	**Arm Staple 90**	
TAGTCTTTTCGTATTAGATGATACAGGGAACC	**Arm Staple 91**	
AACATCGCGAAGTATTCCACCCTCATTTTCAGAGGTTTAG	**Arm Staple 92**	
CAGCAGAAAGCCGTCAAAATATCAAACCCTCATAGCCCGG	**Arm Staple 93**	
CAGCCCTCTCTGAATTAGTTTGAGTAACATTGAAAAAGA	**Arm Staple 94**	
TACAAACTTGGCTTTTAATCCTTTGCCCGAATTAAATTT	**Arm Staple 95**	
CCATGTACCAGAGCCAAGACTTTACAAACAATGGCGGTTG	**Arm Staple 96**	

TCTAAAGCATCACTAGATACCGAAACATTCTGCGGCCTTG	**Arm Staple 97**	
AGACGACGCATTATTACAGGTAGAAAGATTCGTCAGTGC	**Arm Staple 98**	
CAATTACCCAAATCAACGTAACAAATCTACGTTAATAAAA	**Arm Staple 99**	
ATTCAGTGTTCATCAATCGCCTGATAAATTGTCTTGCAGG	**Arm Staple 100**	
CACCAGAAGTGTACAGTGCTCCATGTTACTTACCACGCAT	**Arm Staple 101**	
TAATTGCGAGCAACCGCAAGAATTTGCCGCCAGCAGTTGTCGA	**Arm Staple 102**	
TCGGGAAATAGAACGTCAGCGTGG	**Arm Staple 103**	
AATCGGCCGCACATCCTCATAACGAGGCGGCCTTTAGTGATAGA	**Arm Staple 104**	
CAACAATGCGCGTGAGTTTCTTGCGAATCGAAAGACAGCATCGGCAGCGATT	**Arm Staple 105**	
TTTACATTAAAACAAGCCCATCCAAAAAGGCCGCTT	**Arm Staple 106**	
TTAGGATAAAACAGCAGCAATTGTATCGTATTCGGT	**Arm Staple 107**	
GACGCAGAACGCAACCAGCTTACGCCGGAAGATAAATCA	**Arm Staple 108**	
CTGCTCATGCCAACGGCAGCACCGCCTAATGAGATGGTG	**Arm Staple 109**	
TGTACATCTGCTGGTCTGGTCAGCTTGCGCTCCTGGTTTG	**Arm Staple 110**	
GACATAAAAAAATCCCAGGA	**Arm Staple 111**	
AGCCGCACGAACGTGCCGGACTTGCCTGTCGTGGCCCTGA	**Arm Staple 112**	
AAAGTTAAGCAGCCTCCGGCCAGAAACGCGCGGACGGGCA	**Arm Staple 113**	
GATTGCCGTCTAAAATATCTTTAGTTGGCAAATCAACAGTGATAAGTG	**Arm Staple 114**	
ACCCCGGTGCGCAGTCATAGTTAGCAGAAGGATCCTGATTTAAAAGAG	**Arm Staple 115**	
CCTCAAGTGTACTGGTACAGTGCCCGTATAACGGAACAAATAAAAAC	**Arm Staple 116**	
TACCGCCAGCCTATTTCGGAACCTGAAGAAAAAGCTGCTC	**Arm Staple 117**	
TACTCAGGGGACTTGCTGAACCTCATAGATAATACATTTGGTAAAAAA	**Arm Staple 118**	
AATAGGTGAAAGTATTAAGAGGCTGAACTGGCACGAGAAA	**Arm Staple 119**	
TATAAGTAATCAATATTGAGAGCCAGAGGTGAATTCACCACCGCCAGC	**Arm Staple 120**	
GTCATACAACAACGCCTTATTTGAATGGCTAT	**Arm Staple 121**	
GCCACCCTCGTAACACAACTGATACCTGAAAATCACTTG	**Arm Staple 122**	
GAATACCACATTCAACAGCAAACAAGAGAATCTCATATGT	**Arm Staple 123**	
CGAACTAAACAGTTAATGCCCCCTCCCTCAGAACCGCCAC	**Arm Staple 124**	
CCAGTCAGGACGTTGGATTATTCTGAAACATGTATCACCG	**Arm Staple 125**	
CCTTATGCGATTTTAAGAGACTCCTCAAGAGAAGGGTTGA	**Arm Staple 126**	
CTTGAGTAAATAAGTTTTAACGGG	**Arm Staple 127**	
TCTGTCCAGGCCGATTAAAGGGATGACTCCAACGTCAAAG	**Arm Staple 128**	
ATTAACCGGATGATGGCAATTCATTTTCAGCGTCCACAGA	**Arm Staple 129**	
AATACTTCTCGTTAGAATCAGAGCTCATGAACCATCACCC	**Arm Staple 130**	
GTAATAACGCGTAAGAATACGTGGTAAAGGAAGTCACCAG	**Arm Staple 131**	
CCTGAGTATTGCTTTGACGAGCACGGTCGAGGTGCCGTAA	**Arm Staple 132**	
AAACTATGCCAACAGAGATAGAATGAAAATCATAGGAAC	**Arm Staple 133**	
CTGGTAATCTTAATGCGCCGCTACCCTAAAGGGAGCCCCC	**Arm Staple 134**	
CAATATTAGTCACACGACCAGTAAGCCTTTAAATGAAAAA	**Arm Staple 135**	
CATTGCAATCACGCTGCGCGTAACGGGAAAGC	**Arm Staple 136**	
ACGCTCATGAAATGGATTATTTACTCGAGGTG	**Arm Staple 137**	
CTCAGCAGAATAATTTTTTCACGTCCCTTCTGAGCCCTAA	**Arm Staple 138**	
TTGCGGGAACGGAGATTTGTATCAGAGTAATCTTGACAAG	**Arm Staple 139**	

GAGTTAAAAAAGGCTCCAAAAGGATAAAAGGGCGAACCAC	**Arm Staple 140**	
CGCTGAGGGTCGAAATCCGCGACCACCAGGCGCATAGGCT	**Arm Staple 141**	
GAGAGGCTACAGAGGCTTTGAGAATACACTAAAACGAGGGGGT	**Arm Staple 142**	
AAGGAACAACCACAGACAATATTTCATTGTAGC	**Arm Staple 143**	
AATTTCTTTGACAACAACCATCGCGCCGGAACGAGGCGCAACTTTGAA	**Arm Staple 144**	
AATAGTAAAATGTTTAAGGCATAGTAAGAGCAAGATTTAG	**Arm Staple 145**	
GCCAACTCATCTTTGACCCCAACGAGGGTAGCAACATAGA	**Arm Staple 146**	
AACCGGATATTCAAATCGCGAAACAAAGTACATCGTCACC	**Arm Staple 147**	
GGCTGACCAATAAGGCTTGCCCTGTCATTATA	**Arm Staple 148**	
AGAGGACAGATGAACGCGAGTAGTAAATTGGGGTGAATTA	**Arm Staple 149**	
GAGGCAAAAGGACTAAAGACTTTTAAACAACTTTCAACAGCAAT	**Arm Staple 150**	
ATACCAAGAGCGAGAGGCTTTTGCAAAAGAAGCGTTTACC	**Arm Staple 151**	
AAAGAATAAGAACGTGTTTAGACAGGAACGGTCAGTGAGGCCACCGAGGTTTGGAT	**Arm Staple 152**	
GTTCCGAAACCGTCTAGGGAGCTAAACAGGATCACGCAA	**Arm Staple 153**	
CCCCAGCATTTTTTGGGTATAACGTGCTTTCCTTTGATTA	**Arm Staple 154**	
GAGAGTTGATCGGAACAGGGCGCGTACTATGGGAAGAACTC	**Arm Staple 155**	
ACAGCTGAGCTTGACGCACCACACCCGCCGCGATCCAGAA	**Arm Staple 156**	
**TTTTTT**AGCGGGCGCTAGGGCGGGAAGAAAGCGAAAGG**TTTTTT**	**Arm Staple 157**	
**TTTTTT**TGACGCTCAATCGTCTGGAAATACCTACATTT**TTTTTT**	**Arm Staple 158**	
AACCGATAGTTTATCAGCTTGCTTATTGGCAGGGCGGTCAGTATTAACACC**TTTTTT**	**Arm Staple 159**	
**TTTTTT**ATAGTTGCGCCGACAAAAACAGCTTGATACCG**TTTTTT**	**Arm Staple 160**	
**TTTTTT**GGAACCGAACTGACCAGACGGTCAATCATAAG**TTTTTT**	**Arm Staple 161**	
**TTTTTT**CAGGGTGGTTTAAAGGAAGCTGGCAAGTGTAGCGGCAGGAAAA	**Arm Staple 162**	
**TTTTTT**GCCTGCAACAGTGCCACGCCTGGTCAGGAGCACTAACAACTAATGAAGGGT	**Arm Staple 163**	
**TTTTTT**GCGGTCCGTTTTTTCGTCTCGTCGCTGACGATGCT	**Arm Staple 164**	
CGGTTTGCGTATTGGGCGC**TTTTTT**	**Arm Staple 165**	
**TTTTTT**GAGGAAGGTTATTCCGGCAAAC**TTTTTT**	**Arm Staple 166**	
CCGTCGAGAGGATTAGGATTAGCGGGGTTTTGCTC**TTTTTT**	**Arm Staple 167**	
**TTTTTT**AGTACCAGGCGTGAAAGGAATT**TTTTTT**	**Arm Staple 168**	
**TTTTTT**TGGTGCTGCGGCCAGAATGCGGCGGGCAGTGTCAC	**Arm Staple 169**	
**TTTTTT**TTAAGTTGGGTTGTGCACTCTG**TTTTTT**	**Arm Staple 170**	
**TTTTTT**AGATGGGCGCACTGCAAGGCGA**TTTTTT**	**Arm Staple 171**	
**TTTTTT**TTTAGAACCCTCACGTTGGTGT**TTTTTT**	**Arm Staple 172**	
**TTTTTT**CAAATCCAGGGATGTGTCGTAACC	**Arm Staple 173**	
**TTTTTT**ATAATGCTGTAGCTCAACATGTTTTAAAT	**Arm Staple 174**	
**TTTTTT**GTACCGCACTCATCGAACGGGTATTAAACCAA**TTTTTT**	**Arm Staple 175**	
**TTTTTT**GAATTAACTGAACACCAGCGCATTAGACGGGA**TTTTTT**	**Arm Staple 176**	
TGAAATAGCAATAGCTCAGATAGCTGCTGATG**TTTTTT**	**Arm Staple 177**	
**TTTTTT**TTTTAAGAAAAGTAAGATCTTACCGAAGCCCT**TTTTTT**	**Arm Staple 178**	
AGCAAAATGCGGATGGCTTAGAGCTTAATTGCTGAAT**TTTTTT**	**Arm Staple 179**	
**TTTTTT**TTGAGCCATTTGGGAATATCACCGTCACCGAC**TTTTTT**	**Arm Staple 180**	

TCACTGTTGCCCTTCTCCGTGGTGAA**TTTTTT**	**Arm Staple 181**	
**TTTTTT**ATTTGAATTACCTTAAATCCTCATTA**TTTTTT**	**Arm Staple 182**	
CATTAAATTTTTGCTATCAGGTCAT**TTTTTT**	**Arm Staple 183**	
**TTTTTT**GGCATCAATTCTACTAATAGTAGTAGCATGAGAGATC	**Arm Staple 184**	
**TTTTTT**AGTACCGACAAAAGGTAAAGTAATTCTTGCTA	**Arm Staple 185**	
GACGACAAAATTGTTATCCGCTCACAATT**TTTTTT**	**Arm Staple 186**	
**TTTTTT**CCAGAACCACCACCAGAGCCGCCGCCATCAGAGCCACCGGAAC	**Arm Staple 187**	
TTTTGCACATAAGAGAATATAA**TTTTTT**	**Arm Staple 188**	
**TTTTTT**CATTTTCGAGCCAGTACCAGCTACAATTT	**Arm Staple 189**	
TCAGATGAATATACAGTAACAACATGTAATTTAGGCAGAGG**TTTTTT**	**Arm Staple 190**	
CTCAGAACGGAGAAACTTAATTACATTTAACAATTTC**TTTTTT**	**Arm Staple 191**	
**TTTTTT**ATCACCGGAACCAGAGCCACCACCACCCTCAGAGCCGCCA**TTTTTT**	**Arm Staple 192**	
ACCCTGACTATTATAGTCAGATGCCATCTTTTCATAATCAAA**TTTTTT**	**Arm Staple 193**	
TCAAAAAGTGGGGCGCGAGCTGAAAAGGT**TTTTTT**	**Arm Staple 194**	
GGCGAAAAATCGGCAAAATCCCTTCATAAAGTGTAAAGCC	**Strut Replacement Staple 1**	
ATAAGCGGAATTATCATCATATTTTAAATACCGTTC	**Strut Replacement Staple 2**	
TGGGGTGTCGGTGGTGCCATCCCAACAGCGG	**Strut Replacement Staple 3**	
ATCAAACCGTTATTAATTCCTGATGTAGCATGAGT	**Strut Replacement Staple 4**	
CAGTAAGCAAAACTAGCATGTCAAGATGAACG	**Strut Replacement Staple 5**	
GTAATCGTATCAGTTGACACTATCATAACCCTTTTT	**Strut Replacement Staple 6**	
GCTGTTTCAAAGGTTTCTTTGCTACCAGTCC	**Strut Replacement Staple 7**	
CGGAATTTTACATAAACATCAAGATCAGACGACAACATAT	**Strut Replacement Staple 8**	
TTGGCCTTTTTAACCAATAGGAAGAGGGTAG	**Strut Replacement Staple 9**	
CTATTTTTTAACATCTAGCTATATTTTCATTATTAAGAG	**Strut Replacement Staple 10**	
GCTAATGCAGAACGCGGTAATCATGGTCATA	**Strut Replacement Staple 11**	
AAGATGATGAAACAAATCAATATAAGAATCCT	**Strut Replacement Staple 12**	
TTTTTTTTCAACTTTAATCATTCTTGAGATGGTTTAATTTTTTT	**Biotin Anchor Replacement Staple 1**	
TTAAATGCCTTTATTTCAACGCAAGGATAAAAATTTTTTTT	**Biotin Anchor Replacement Staple 2**	

Table S2 nDFS Staples that bind on scaffold linkers in various design versions.

**Sequence**	**Element**	
AATCCGCCGGGCGCGGTTGCGGTATGAGTCAGCGGGGTCATTGCAGGCGCTTTCGC	**nDFS.B Staples 1**	
AATAAAGAAATTGCGTAGATTTTCAGGTACCATATCAAAATTATTTGCACGTAAAA	**nDFS.B Staples 2**	
AAATTGTAAACGTTAATATTTTGTTAAACAAAAACAGGAAGATTGTATAAGCAAAT	**nDFS.B Staples 3**	
CGAGAATGACCATAAATCAAAAATCAGGTCCCCCTCAAATGCTTTAAACAGTTCAG	**nDFS.B Staples 4**	
**TTTTTT**ACACAACATACGAGGCTGGAGGTGTCCAGCA	**nDFS.C-5 Staples 1**	
**TTTTTT**GCCAGAATGGAAATGATAATCAGAAAAGCCC	**nDFS.C-5 Staples 2**	
CCGGGTCACTGTTGCCCTTCTCCGTGGTGAA**TTTTTT**	**nDFS.C-5 Staples 3**	
ATTCGCATTAAATTTTTGCTATCAGGTCAT**TTTTTT**	**nDFS.C-5 Staples 4**	
TTAACGTCAGATGAATATACAGTAACAACATGTAATTTAGGCAGAGG**TTTTTT**	**nDFS.C-5 Staples 5**	
TCTTTACCCTGACTATTATAGTCAGATGCCATCTTTTCATAATCAAA**TTTTTT**	**nDFS.C-5 Staples 6**	
AAGTTTCCGAAGGCACCAACCTAAGCGTCCAATACTGCGGAATCGTCATAAATATTC ATTGAA	**nDFS.C-5 Staples 7**	
**TTTTTT**GTTAGTAAATGAATTTTCTTGAATAATGGAAGGGTTAGAACCT	**nDFS.C-5 Staples 8**	
**TTTTTT**ACACAACATACGAGGCTGGAGGTGTCCAGCATCAGC	**nDFS.C-10 Staples 1**	
**TTTTTT**GCCAGAATGGAAATGATAATCAGAAAAGCCCCAAAA	**nDFS.C-10 Staples 2**	
ATGAGCCGGGTCACTGTTGCCCTTCTCCGTGGTGAA**TTTTTT**	**nDFS.C-10 Staples 3**	
TTAAAATTCGCATTAAATTTTTGCTATCAGGTCAT**TTTTTT**	**nDFS.C-10 Staples 4**	
CAGGTTTAACGTCAGATGAATATACAGTAACAACATGTAATTTAGGCAGAGG**TTTTT T**	**nDFS.C-10 Staples 5**	
TCAGGTCTTTACCCTGACTATTATAGTCAGATGCCATCTTTTCATAATCAAA**TTTTTT**	**nDFS.C-10 Staples 6**	
AAGTTTCCGAAGGCACCAACCTAAGCGTCCAATACTGCGGAATCGTCATAAATATTC ATTGAATCCCC	**nDFS.C-10 Staples 7**	
**TTTTTT**GTTAGTAAATGAATTTTCTTGAATAATGGAAGGGTTAGAACCTACCAT	**nDFS.C-10 Staples 8**	
**TTTTTT**ACACAACATACGAGGCTGGAGGTGTCCAGCATCAGCGGGGT	**nDFS.C-15 Staples 1**	
**TTTTTT**GCCAGAATGGAAATGATAATCAGAAAAGCCCCAAAAACAGG	**nDFS.C-15 Staples 2**	

GCGGTATGAGCCGGGTCACTGTTGCCCTTCTCCGTGGTGAA**TTTTTT**	**nDFS.C-15 Staples 3**	
TTTTGTTAAAATTCGCATTAAATTTTTGCTATCAGGTCAT**TTTTTT**	**nDFS.C-15 Staples 4**	
ATTTTCAGGTTTAACGTCAGATGAATATACAGTAACAACATGTAATTTAGGCAGAGG **TTTTTT**	**nDFS.C-15 Staples 5**	
AAAAATCAGGTCTTTACCCTGACTATTATAGTCAGATGCCATCTTTTCATAATCAAA**T TTTTT**	**nDFS.C-15 Staples 6**	
AAGTTTCCGAAGGCACCAACCTAAGCGTCCAATACTGCGGAATCGTCATAAATATTC ATTGAATCCCCCTCAA	**nDFS.C-15 Staples 7**	
**TTTTTT**GTTAGTAAATGAATTTTCTTGAATAATGGAAGGGTTAGAACCTACCATATCA A	**nDFS.C-15 Staples 8**	
**TTTTTT**ACACAACATACGAGGCTGGAGGTGTCCAGCATCAGCGGGGTCATTG	**nDFS.C-20 Staples 1**	
**TTTTTT**GCCAGAATGGAAATGATAATCAGAAAAGCCCCAAAAACAGGAAGAT	**nDFS.C-20 Staples 2**	
CGGTTGCGGTATGAGCCGGGTCACTGTTGCCCTTCTCCGTGGTGAA**TTTTTT**	**nDFS.C-20 Staples 3**	
TAATATTTTGTTAAAATTCGCATTAAATTTTTGCTATCAGGTCAT**TTTTTT**	**nDFS.C-20 Staples 4**	
CGTAGATTTTCAGGTTTAACGTCAGATGAATATACAGTAACAACATGTAATTTAGGC AGAGG**TTTTTT**	**nDFS.C-20 Staples 5**	
AAATCAAAAATCAGGTCTTTACCCTGACTATTATAGTCAGATGCCATCTTTTCATAAT CAAA**TTTTTT**	**nDFS.C-20 Staples 6**	
AAGTTTCCGAAGGCACCAACCTAAGCGTCCAATACTGCGGAATCGTCATAAATATTC ATTGAATCCCCCTCAAATGCT	**nDFS.C-20 Staples 7**	
**TTTTTT**GTTAGTAAATGAATTTTCTTGAATAATGGAAGGGTTAGAACCTACCATATCA AAATTA	**nDFS.C-20 Staples 8**	
**TTTTTT**ACACAACATACGAGGCTGGAGGTGTCCAGCATCAGCGGGGTCATTGCAGG C	**nDFS.C-25 Staples 1**	
**TTTTTT**GCCAGAATGGAAATGATAATCAGAAAAGCCCCAAAAACAGGAAGATTGTA T	**nDFS.C-25 Staples 2**	
GGGCGCGGTTGCGGTATGAGCCGGGTCACTGTTGCCCTTCTCCGTGGTGAA**TTTTTT**	**nDFS.C-25 Staples 3**	
AACGTTAATATTTTGTTAAAATTCGCATTAAATTTTTGCTATCAGGTCAT**TTTTTT**	**nDFS.C-25 Staples 4**	
AATTGCGTAGATTTTCAGGTTTAACGTCAGATGAATATACAGTAACAACATGTAATT TAGGCAGAGG**TTTTTT**	**nDFS.C-25 Staples 5**	
ACCATAAATCAAAAATCAGGTCTTTACCCTGACTATTATAGTCAGATGCCATCTTTTC ATAATCAAA**TTTTTT**	**nDFS.C-25 Staples 6**	
AAGTTTCCGAAGGCACCAACCTAAGCGTCCAATACTGCGGAATCGTCATAAATATTC ATTGAATCCCCCTCAAATGCTTTAAA	**nDFS.C-25 Staples 7**	
**TTTTTT**GTTAGTAAATGAATTTTCTTGAATAATGGAAGGGTTAGAACCTACCATATCA AAATTATTTGC	**nDFS.C-25 Staples 8**	

**TTTTTT**ACACAACATACGAGGCTGGAGGTGTCCAGCATCAGCGGGGTCATTGCAGGCGCTTT	**nDFS.C-30 Staples 1**	
**TTTTTT**GCCAGAATGGAAATGATAATCAGAAAAGCCCCAAAAACAGGAAGATTGTATAAGCA	**nDFS.C-30 Staples 2**	
CCGCCGGGCGCGGTTGCGGTATGAGCCGGGTCACTGTTGCCCTTCTCCGTGGTGAA**TTTTTT**	**nDFS.C-30 Staples 3**	
TTGTAAACGTTAATATTTTGTTAAAATTCGCATTAAATTTTTGCTATCAGGTCAT**TTTTTT**	**nDFS.C-30 Staples 4**	
AAAGAAATTGCGTAGATTTTCAGGTTTAACGTCAGATGAATATACAGTAACAACATGTAATTTAGGCAGAGG**TTTTTT**	**nDFS.C-30 Staples 5**	
GAATGACCATAAATCAAAAATCAGGTCTTTACCCTGACTATTATAGTCAGATGCCATCTTTTCATAATCAAA**TTTTTT**	**nDFS.C-30 Staples 6**	
AAGTTTCCGAAGGCACCAACCTAAGCGTCCAATACTGCGGAATCGTCATAAATATTCATTGAATCCCCCTCAAATGCTTTAAACAGTT	**nDFS.C-30 Staples 7**	
**TTTTTT**GTTAGTAAATGAATTTTCTTGAATAATGGAAGGGTTAGAACCTACCATATCAAAATTATTTGCACGTA	**nDFS.C-30 Staples 8**	
**TTTTTT**ACACAACATACGAGGCTGGAGGTGTCCAGCATCAGCGGGGTCATTGCAGGCGCTTTCGCA	**nDFS.C-34 Staples 1**	
**TTTTTT**GCCAGAATGGAAATGATAATCAGAAAAGCCCCAAAAACAGGAAGATTGTATAAGCAAATA	**nDFS.C-34 Staples 2**	
CAATCCGCCGGGCGCGGTTGCGGTATGAGCCGGGTCACTGTTGCCCTTCTCCGTGGTGAA**TTTTTT**	**nDFS.C-34 Staples 3**	
TAAATTGTAAACGTTAATATTTTGTTAAAATTCGCATTAAATTTTTGCTATCAGGTCAT**TTTTTT**	**nDFS.C-34 Staples 4**	
AAATAAAGAAATTGCGTAGATTTTCAGGTTTAACGTCAGATGAATATACAGTAACAACATGTAATTTAGGCAGAGG**TTTTTT**	**nDFS.C-34 Staples 5**	
ACGAGAATGACCATAAATCAAAAATCAGGTCTTTACCCTGACTATTATAGTCAGATGCCATCTTTTCATAATCAAA**TTTTTT**	**nDFS.C-34 Staples 6**	
AAGTTTCCGAAGGCACCAACCTAAGCGTCCAATACTGCGGAATCGTCATAAATATTCATTGAATCCCCCTCAAATGCTTTAAACAGTTCAGA	**nDFS.C-34 Staples 7**	
**TTTTTT**GTTAGTAAATGAATTTTCTTGAATAATGGAAGGGTTAGAACCTACCATATCAAAATTATTTGCACGTAAAAC	**nDFS.C-34 Staples 8**	
**TTTTTT**ACACAACATACGAGGCTGGAGGTGTCCAGCATCAGCGGGGTCATTGCAGGCGCTTTCGCAC	**nDFS.C-35 Staples 1**	
**TTTTTT**GCCAGAATGGAAATGATAATCAGAAAAGCCCCAAAAACAGGAAGATTGTATAAGCAAATAT	**nDFS.C-35 Staples 2**	
TCAATCCGCCGGGCGCGGTTGCGGTATGAGCCGGGTCACTGTTGCCCTTCTCCGTGGTGAA**TTTTTT**	**nDFS.C-35 Staples 3**	
TTAAATTGTAAACGTTAATATTTTGTTAAAATTCGCATTAAATTTTTGCTATCAGGTCAT**TTTTTT**	**nDFS.C-35 Staples 4**	
GAAATAAAGAAATTGCGTAGATTTTCAGGTTTAACGTCAGATGAATATACAGTAACAACATGTAATTTAGGCAGAGG**TTTTTT**	**nDFS.C-35 Staples 5**	
AACGAGAATGACCATAAATCAAAAATCAGGTCTTTACCCTGACTATTATAGTCAGATGCCATCTTTTCATAATCAAA**TTTTTT**	**nDFS.C-35 Staples 6**	

AAGTTTCCGAAGGCACCAACCTAAGCGTCCAATACTGCGGAATCGTCATAAATATTC ATTGAATCCCCCTCAAATGCTTTAAACAGTTCAGAA	**nDFS.C-35 Staples 7**	
**TTTTTT**GTTAGTAAATGAATTTTCTTGAATAATGGAAGGGTTAGAACCTACCATATCA AAATTATTTGCACGTAAAACA	**nDFS.C-35 Staples 8**	

Table S3 Other nDFS staples

**Staple Sequence**	**Element**	
ACTCCCACCTATCACTCGCCCGCATTAACTAAGACA	**Opening Strand Staple 1**	
CTAGGGGTTATGCTGGCACACATTTGATCACTATGC	**Opening Strand Staple 2**	
TGTCTTAGTTAATGCGGGCGAGTGATAGGTGGGAGT	**Closing Strand Staple 1**	
GCATAGTGATCAAATGTGTGCCAGCATAACCCCTAG	**Closing Strand Staple 2**	
[Btn]**TTTTTT**TTCAACTTTAATCATTCTTGAGATGGTTTAAT**TTTTTT**	**Biotin Anchor Staple 1**	
TTAAATGCCTTTATTTCAACGCAAGGATAAAAATT**TTTTTT** [Btn]	**Biotin Anchor Staple 2**	
GGCGAAAAATCGGCAAAATCCCTTCATAAAGTGTAAAGCCTTCGCATTAACTAA GACAGAT	**Strut Staple 1**	
CAGTAAGCAAAACTAGCATGTCAAGATGAACGTTCATTTGATCACTATGCCTA	**Strut Staple 2**	
GCTAATGCAGAACGCGGTAATCATGGTCATATTATCTGTCTTAGTTAATGCGGGC GAGTGAT	**Strut Staple 3**	
TTGGCCTTTTTAACCAATAGGAAGAGGGTAGTTTAGGCATAGTGATCAAATGTG TGCCAGCA	**Strut Staple 4**	

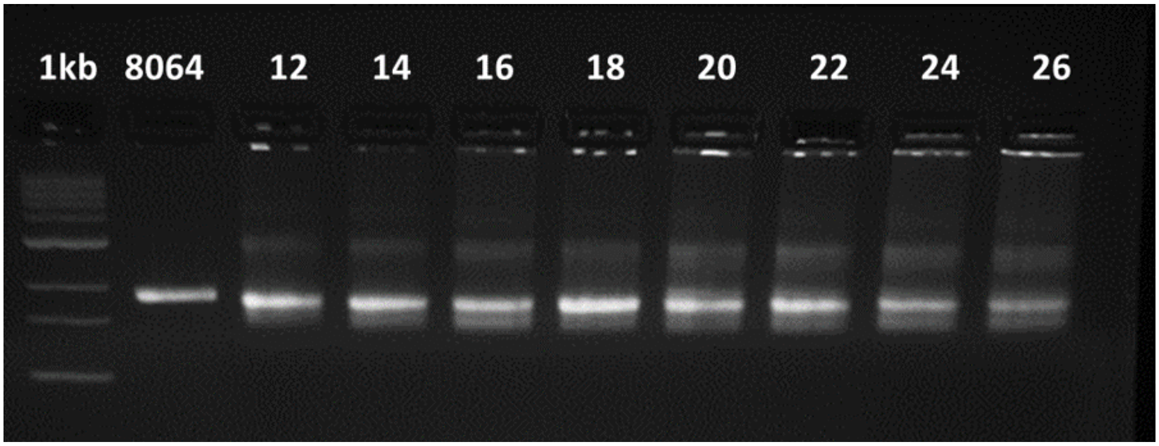

Figure S2 Agarose gel showing nDFS.A folded in [12,14,16,18,20,22,24,26] mM MgCl2 with 1kb ladder and p8064 scaffold as a control. 18 mM $MgCl_2$ was chosen for all subsequent folding reactions.

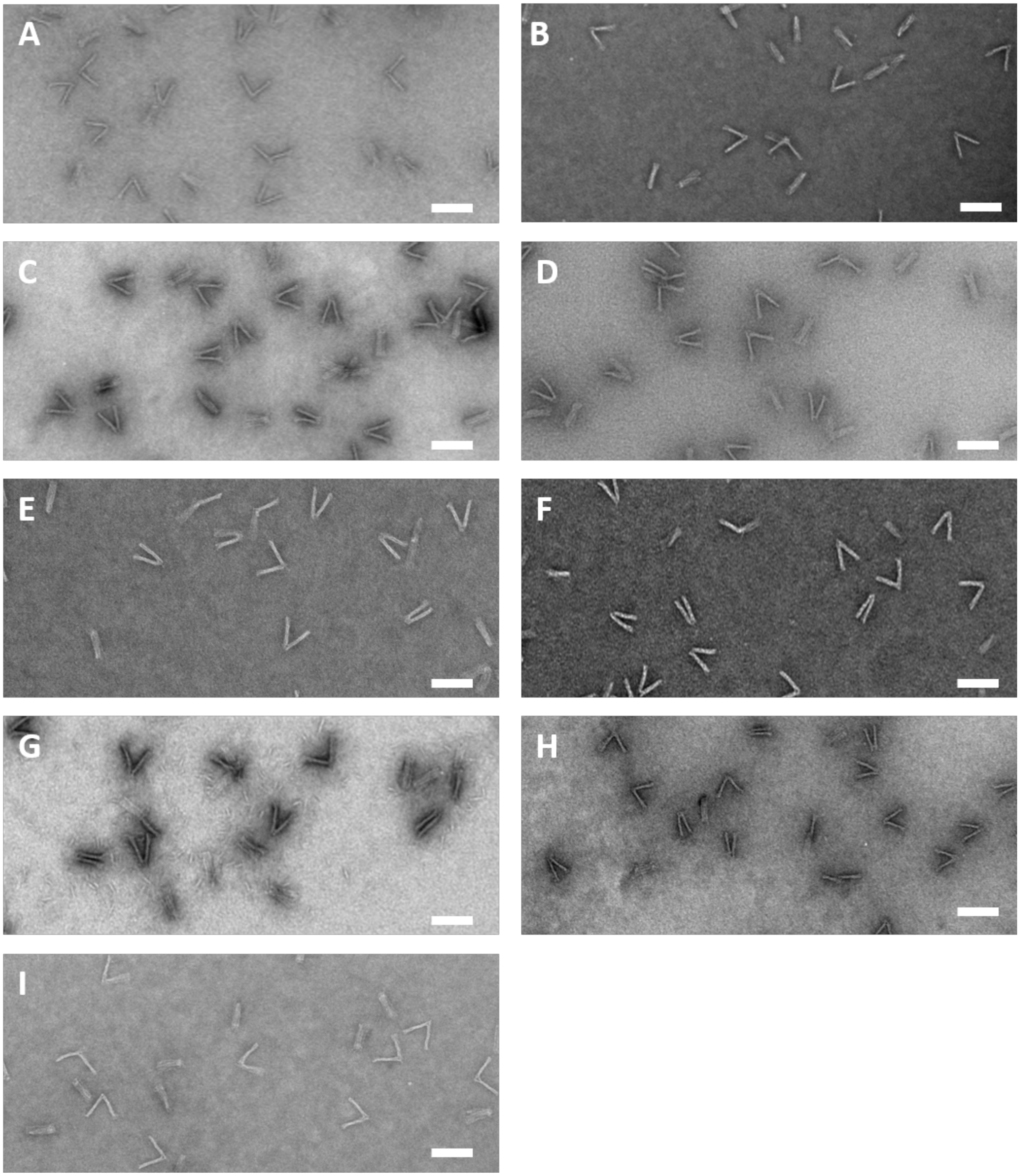

Figure S3 Representative TEM images of (A) nDFS.C-5, (B) nDFS.C-10, (C) nDFS.C-15, (D) nDFS.C-20, (E) nDFS.C-25, (F) nDFS.C-30, (G) nDFS.C-34, (H) nDFS.C-35, (I) nDFS.B. (Scale bars = 100 nm).

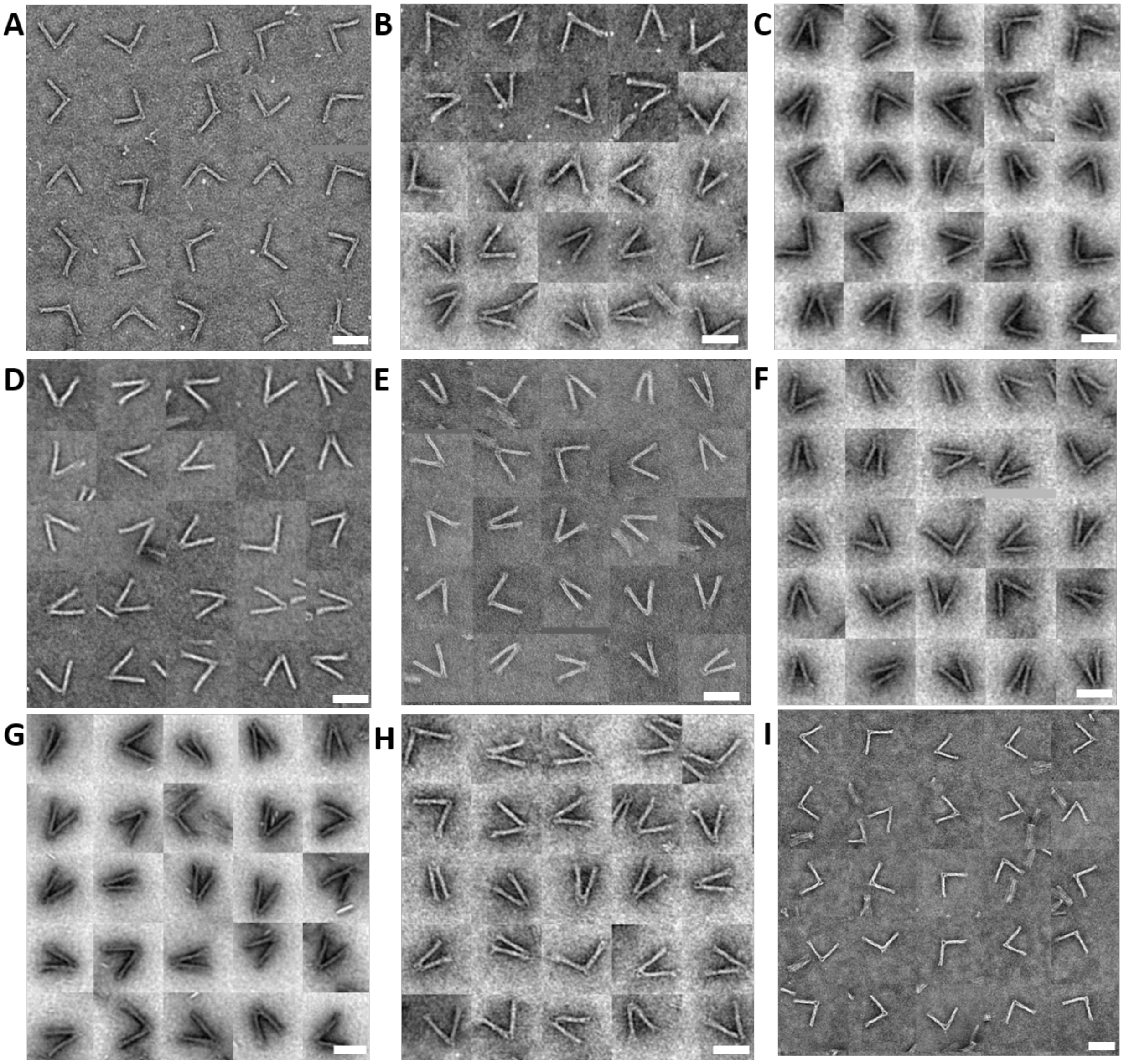

Figure S4 Sample TEM image galleries of (A) nDFS.C-5, (B) nDFS.C-10, (C) nDFS.C-15, (D) nDFS.C-20, (E) nDFS.C-25, (F) nDFS.C-30, (G) nDFS.C-34, (H) nDFS.C-35, (I) nDFS.B. (Scale bars = 60 nm).

Table S4 Sample Sizes in TEM Characterization

Device	Total Sample Size, N
nDFS.A	707 from 3 repeats
nDFS.B	938 from 3 repeats
nDFS.C-5	493 from 2 repeats
nDFS.C-10	575 from 2 repeats
nDFS.C-15	662 from 2 repeats
nDFS.C-20	750 from 3 repeats
nDFS.C-25	729 from 3 repeats
nDFS.C-30	621 from 2 repeats
nDFS.C-34	633 from 2 repeats
nDFS.C-35	595 from 2 repeats
nDFS.B Folded Close	554 from 3 repeats
nDFS.B Toggled Open	348 from 2 repeats
nDFS.B Toggled Close	292 from 2 repeats
nDFS.B Folded Open w/DNA	234 from 2 repeats
nDFS.B Toggled Close w/DNA	252 from 2 repeats
nDFS B with Nucleosome	242 from 3 repeats
nDFS C-35 with Nucleosome	276 from 3 repeats

Table S5 249 bp dsDNA sequence

AGCTTGTCGACGAATTCAGATTCATAAGGAGGACACTGGGACATGCATCGGCTGGAGACCGGAG<br>GGCTGCCCTCCGGTCAATTGGTCGTAGACAGCTCTAGCACCGCTTAAACGCACGTACGCGCTGTC<br>CCCCGCGTTTTAACCGCCAAGGGGATTACTCCCTAGTCTCCAGGCACGTGTCAGATATATACATC<br>CTGTATAATGCATAGGGCAGTGAGTTGCGCTACAATCA CGAATTCTGGATCCGA

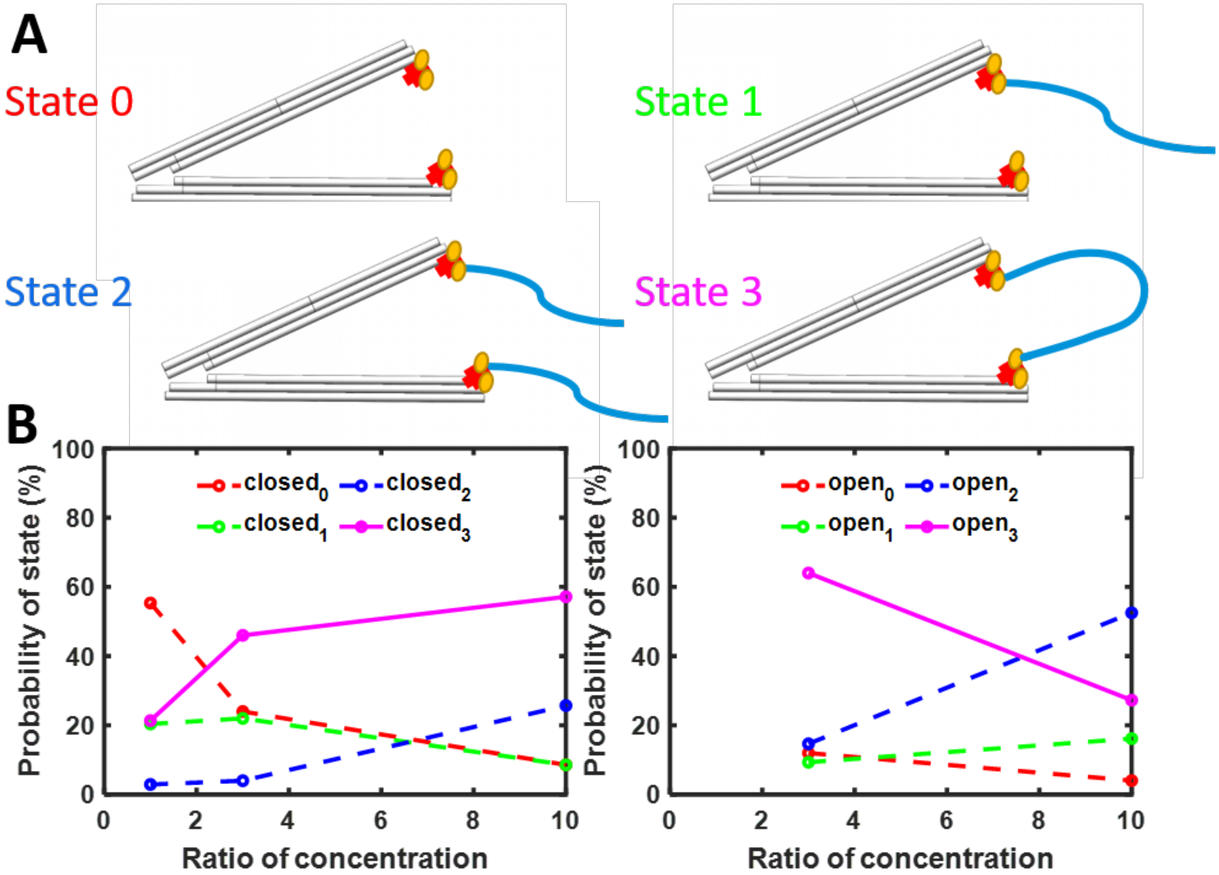

Figure S5 dsDNA binding efficiency to nDFS in both closed and open states quantified by TEM. A) There are 4 possible states upon incorporation of the dsDNA sample. State 0: nDFS incorporates no dsDNA. State 1: nDFS has one dsDNA bound at one of the attachment points. Note this could occur on either arm. We cannot differentiate the two, so we count them as one state. State 2: nDFS has one dsDNA bound at each attachment point. State 3: nDFS correctly incorporates with dsDNA. B) Left plot: for closed nDFS.B, 10-fold excess of the dsDNA sample results in higher binding efficiency of proper incorporation. Right plot: for the open nDFS.B, a 3-fold excess of the dsDNA sample results in higher binding efficiency. Data is quantified via TEM analysis. For the closed hinge, sample sizes for ratio of concentration 1, 3, and 10 are 103, 100, and 105aa. For the opened hinge, sample sizes for ratio of concentration 3 and 10 are 75 and 99, respectively.

Section 1: Additional details on DNA Compression Prediction Model

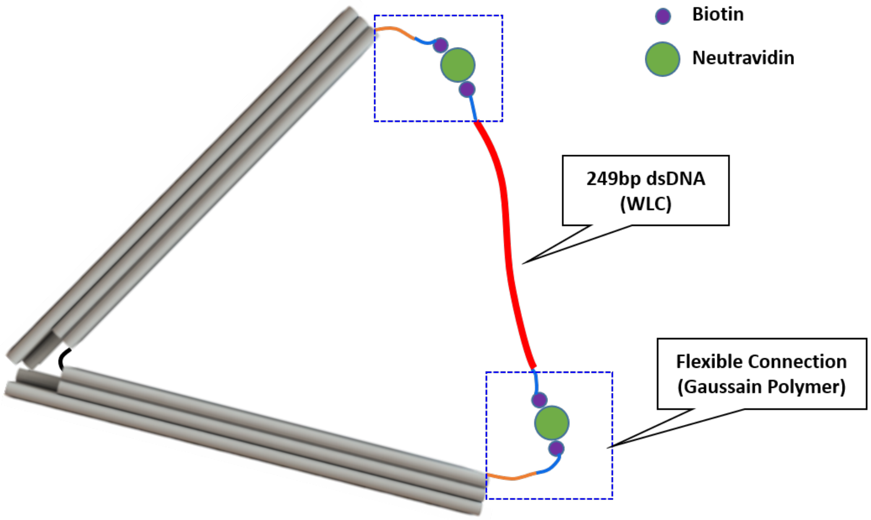

Figure S6 nDFS-dsDNA schematic.

1.1 Confirming that the Frey model is applicable for deformations of interest

Since the Frey formula (1) is an approximation that assumes that a polymer is relatively straight and the hinge introduces bending into the DNA, we check the range of validity of the Frey formula via a comparison of Frey EED distributions to EEDs calculated using the coarse-grained DNA simulation oxDNA (2). In particular, we introduce a mutual trap in oxDNA such that the ends of a simulated version of the linker DNA are attracted to each other (2). This biases the EED distribution towards more compressed configurations, which otherwise would not occur often enough to generate sufficient data. Since the simulated compressive force applied at each EED is known, it can subsequently be divided out to generate a well-sampled EED distribution. Since the simulation provides the dynamical behavior of the molecule, two configurations saved within a small time interval from each other will tend to show similar shapes. In order to compute the equilibrium distribution of the EED, we need to collect independent configurations. Thus, we show the correlation function between the EEDs of consecutive configurations in figure S7A and use it decaying to zero when the two configurations are taken 15 time steps apart as an indication that we will be averaging over independent configurations if we consider only every fifteenth saved configuration. As shown in Figure S7B, the resulting EED distribution agrees with the Frey distribution for extensions greater than 20 nm. Excluding contributions from EEDs <20 nm results in a negligible change in the overall prediction of the angle distribution. Furthermore, we conducted experiments by directly measuring the EED of 147bp dsDNA and it agrees well with Frey model (Figure S7B). Therefore, we conclude that the use of the Frey formula is appropriate in this case.

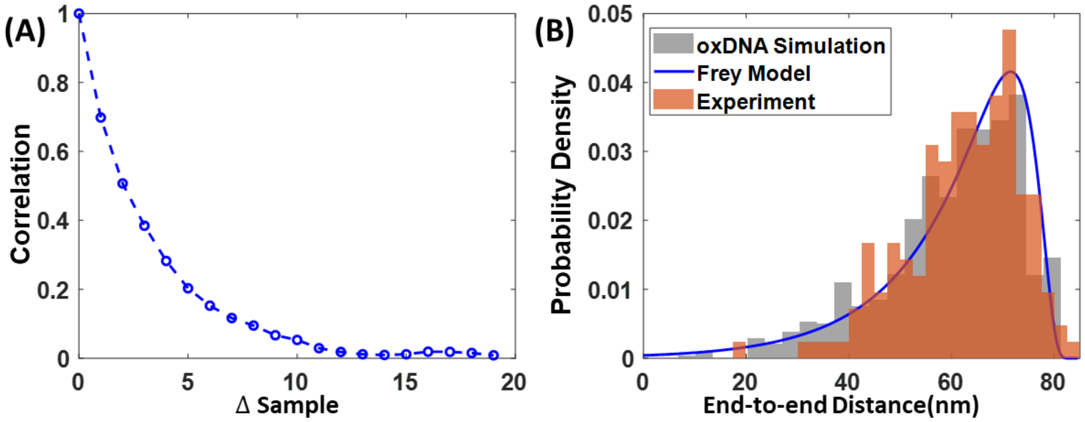

Figure S7 (A) oxDNA correlation test. The correlation function based on temporal distance separating oxDNA simulation samples shows that samples are uncorrelated after 15 sampling intervals. (B) Frey Model validation by oxDNA and experiment.

1.2 Calculation of force

In order to estimate the force exerted by the hinge on the linker DNA, we calculate the WLC model force at the extension corresponding to the peak of the experimental probability distribution. In particular, the force exerted by the hinge as a function of the angle θ is given by

$$F(\theta) = -\frac{\partial G(\theta)}{\partial r} = k_B T \frac{\partial}{\partial r} \ln P(\theta) = k_B T \frac{\partial}{\partial \theta} \ln P(\theta) \frac{\partial \theta}{\partial r} = \frac{k_B T}{P(\theta)} \frac{\partial P(\theta)}{\partial \theta} \frac{\partial \theta}{\partial r} \quad \text{(S1)}$$

where $r = 2L\sin(\theta/2)$ is the distance between the ends of the hinge arms (and also the end-to-end distance of the polymer), and $P(\theta)$ is the probability distribution of the model. Analytically, $\frac{\partial\theta}{\partial r} = \frac{2}{L\sqrt{4-r^2/L^2}}$, but we need to calculate $\frac{\partial P}{\partial \theta}$ numerically. $L = 61$ nm is the length of the hinge arms. Given a set of probabilities P_i for angle bins i, $\frac{\partial P_i}{\partial \theta} = \frac{(P_{i+1}-P_{i-1})}{2\Delta\theta}$where $\Delta\theta$ is the bin size, we can roughly approximate the applied hinge force as the force in the bin corresponding to the peak of the experimental nDFS+dsDNA distribution. This results in a 0.28±0.06pN compression force at the peak of the experimental probability distribution. The error bar is determined by considering different potential persistence lengths of DNA from 40 nm to 60 nm (3).

1.3 Connection assumptions and validation

While we do not have a precise understanding of the length of the connection between the dsDNA and the hinge arms, we know that it is on the order of 10 nm long. We therefore choose nb =10 nm and vary nb by a factor of two to confirm that our calculation is not sensitive to the choice of this parameter. Similarly, since we do not know the precise intrinsic flexibility of the connection, we choose n=2 and vary n by a factor of two in each direction holding nb constant to confirm that our calculation is not sensitive to the choice of this parameter. Finally, while we would expect that the hinge arms exclude some volume that would be otherwise available to the connection, we assume the simplifying case in which the connection is completely free to rotate. We check this assumption in the extreme case that each connection is restricted to the inner half-sphere ($0 < \phi_1 < \pi$ and $\pi < \phi_2 < 2\pi$ in Figure S8). In the closed case, this change somewhat favors more open configurations, but does not change the peak of the angular distribution. In the open case, this also favors more open configurations, shifting the peak by a single bin (and therefore 5°). While connection excluded volume does not seem to produce a large enough effect to explain the observed discrepancy in peak angle between model predictions and experiments quantitatively, the observation that the excluded

volume effect shifts the peak in the correct direction suggests that a more precise treatment of connection excluded volume may increase the predictive power of the model. The experiments necessary to measure this excluded volume, however, are beyond the scope of this paper.

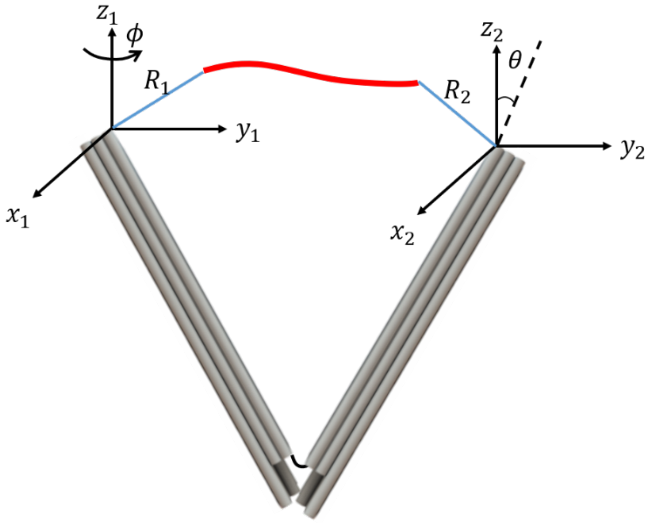

Figure S8 Local spherical coordinate system schematic. The origins are selected at the end of each arm tip where the biotinylated staple strand that the dsDNA sample binds to extends from the arm. ϕ rotates around the z-axis in a right-handed fashion and is zero when it is parallel to the x-axis. θ rotates around the x-axis and is 0 when parallel to the z-axis and π when anti-parallel to the z-axis. The effect of potential excluded volume on the results of the DNA-hinge predictions was tested by recalculating the model predictions under the restriction $0 < \phi_1 < \pi$ and $\pi < \phi_2 < 2\pi$.

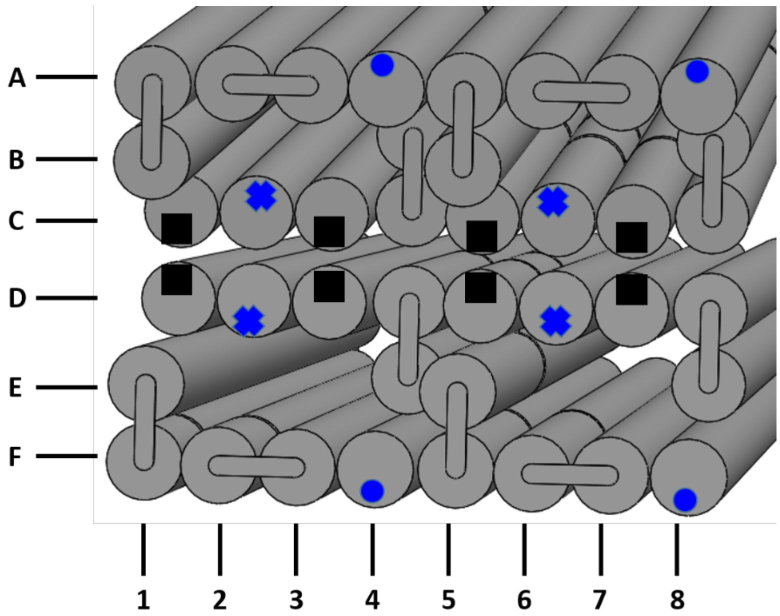

Figure S9 The back view of nDFS bundle model showing details of scaffold routing. Cylinders represent dsDNA helices and the bars that connect the cylinders represent external scaffold connections. Bundles B1, B5, E1, E5 have the same length as layer A and F, while bundles B4, B8, E4, E8 have the same length as layer C and D and are shorter than layers A and F. The locations where scaffold linker connections extend out of the arm are marked with symbols. The blue dots represent the two outer 70 nt connections. The blue crosses represent the two inner 70 nt connections; and the black squares represent the four 2 nt connections.

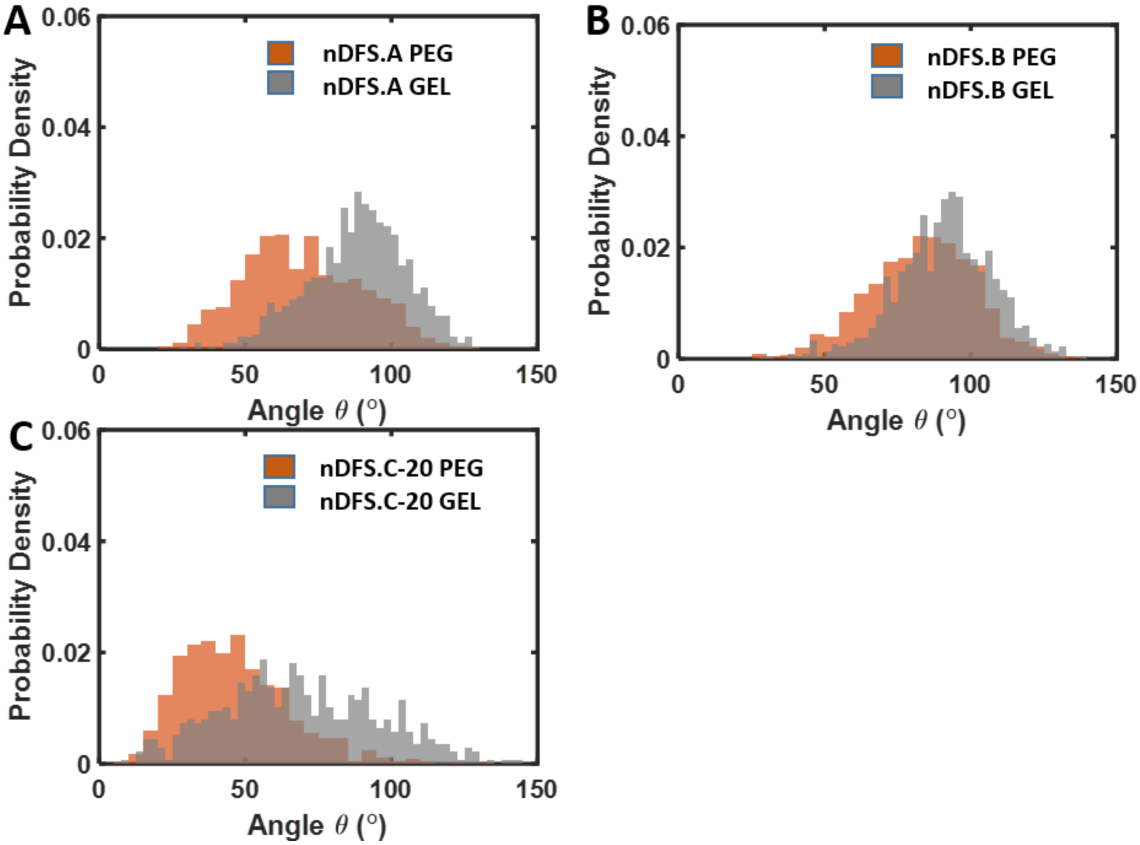

Figure S10. nDFS angular distribution results are influenced by purification method. A) Comparison of PEG-purified and gel-purified structures for nDFS.A. B) Comparison of PEG and gel for nDFS.B. C) Comparison of PEG-purified and gel-purified for nDFS.C-20. Note that nDFS.A and nDFS.C-20 have an obvious distribution shift.

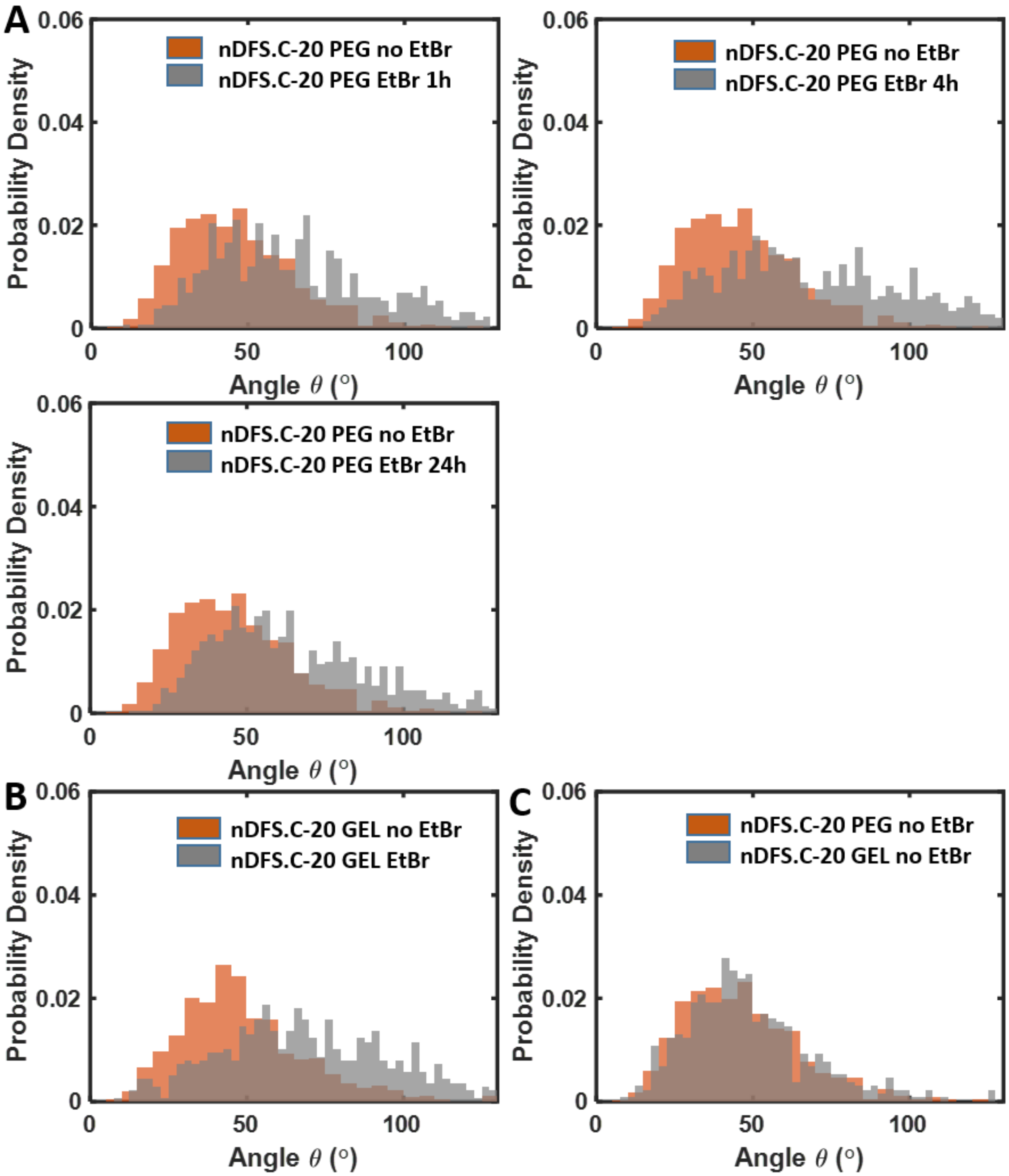

Figure S11 EtBr effect on nDFS.C-20. A) The angular distribution comparison for PEG purification without EtBr and with EtBr for multiple incubation times ([1,4,24] hours). B) The angular distribution comparison for gel purification without EtBr and with EtBr. To perform EtBr-free purification, we ran 3 lanes of the same structure, and the two outer lanes were cut off and stained in 0.005%v/v EtBr for 1 hr. The three gel pieces were then re-aligned to identify the location of the center band for excision. C) The angular distribution comparison for PEG purification without EtBr and gel purification without EtBr. (All EtBr concentrations were the same as gel, 0.005%v/v)

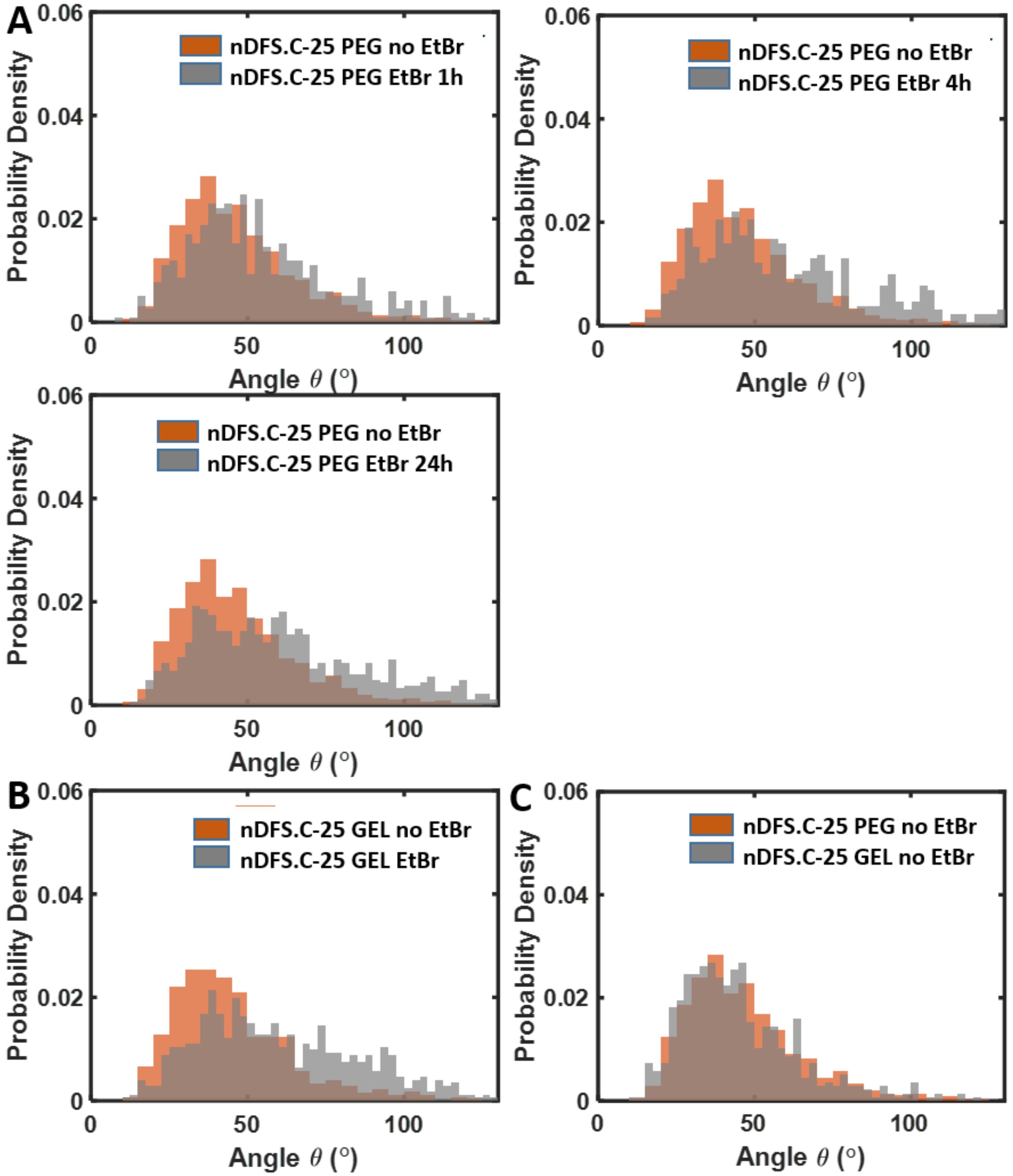

Figure S12 EtBr effect on nDFS.C-25. A) The angular distribution comparison for PEG purification without EtBr and with EtBr for multiple incubation times ([1,4,24] hours). B) The angular distribution comparison for gel purification without EtBr and with EtBr. EtBr-free purification was performed as previously described for nDFS.C-20. C) The angular distribution comparison for PEG purification without EtBr and gel purification without EtBr. (All EtBr concentrations were the same as gel, 0.005%v/v)

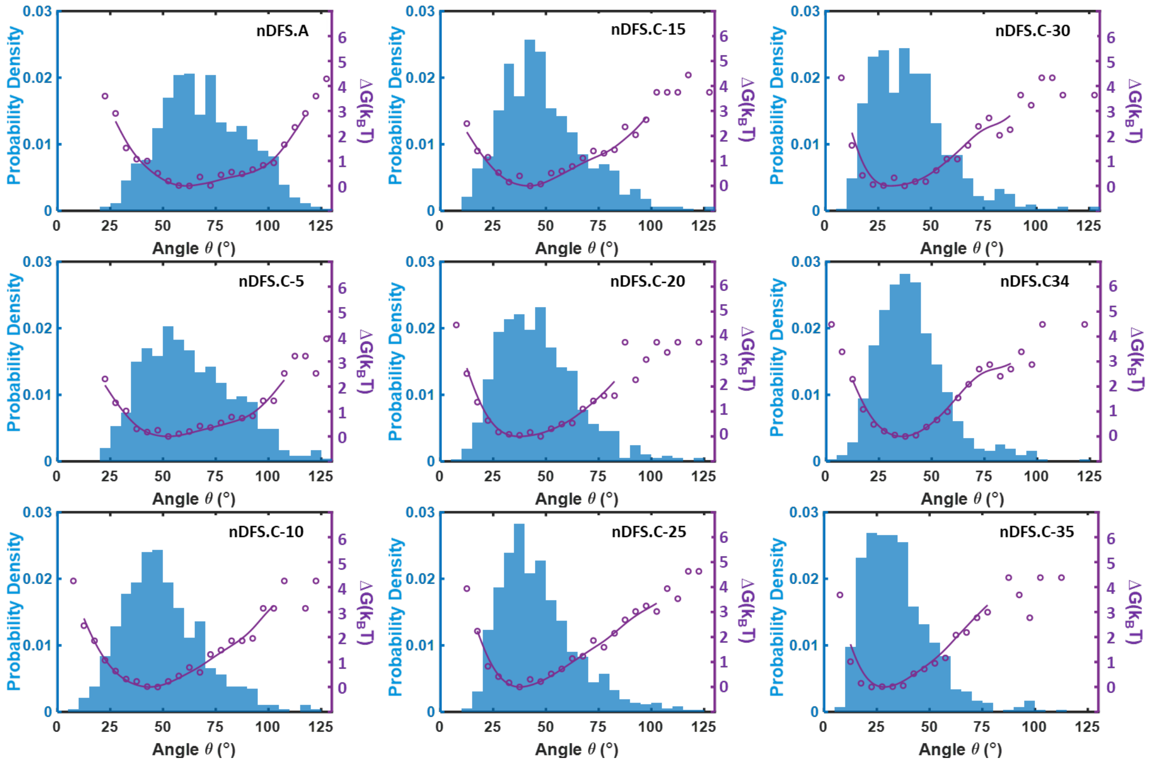

Figure S13 The angular distributions and free energy landscapes of nDFS.C with different lengths of the staples that extend out onto the scaffold linkers. The minimum of the free energy landscape shifts towards smaller angles as the staple length increases. Note: 1) There still exists an angle distribution shift between nDFS.C-34 and nDFS.C-35. 2) nDFS.A is the same as nDFS.C-0.

Table S6 The forces generated by multiple versions of nDFS within limited sampling region. It is likely that the devices could apply even higher forces at the more extreme angles in compression (extreme open angles) or tension (extreme closed angles)

	Max Tensile Force (pN)	At Angle (deg)	Max Compressive Force (pN)	At Angle (deg)
nDFS.A	3.7±0.8	25	2.7±0.5	114
nDFS.B	2.4±0.6	40	2.5±0.4	120
nDFS.C-5	3.0±0.8	20	2.3±0.5	105
nDFS.C-10	3.5±0.9	10	1.6±0.5	100
nDFS.C-15	3.0±0.7	10	2.0±0.5	95
nDFS.C-20	5±1	10	1.9±0.4	80
nDFS.C-25	5±1	15	0.9±0.5	100
nDFS.C-30	5±2	10	1.0±0.5	85
nDFS.C-34	4.3±0.8	10	2.2±0.4	65
nDFS.C-35	5±2	10	1.5±0.6	75

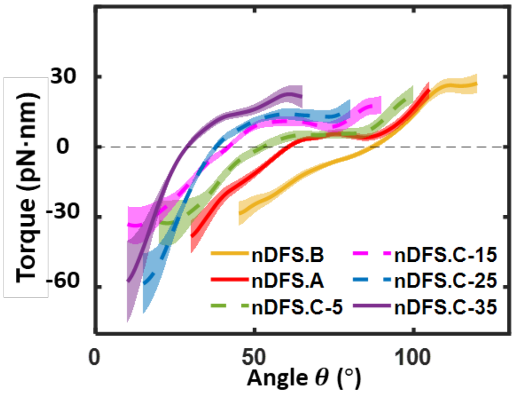

Figure S14 The calculation of torque versus angle for different versions of nDFS based on their free energy landscapes. The error bars are estimated by sample bootstrapping. Note that the error is larger when the angle (and thus the sample count at that angle) is very low or very high.

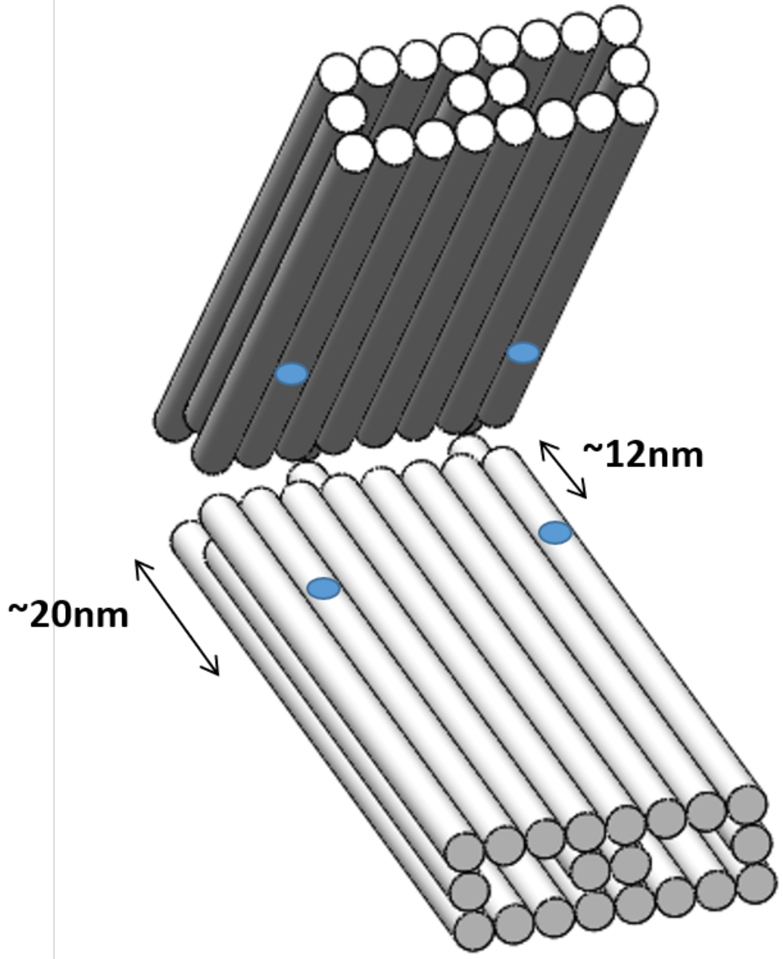

Figure S15 Internal strut locations. Locations of internal struts are indicated by blue dots, 12 nm from the hinge vertex.

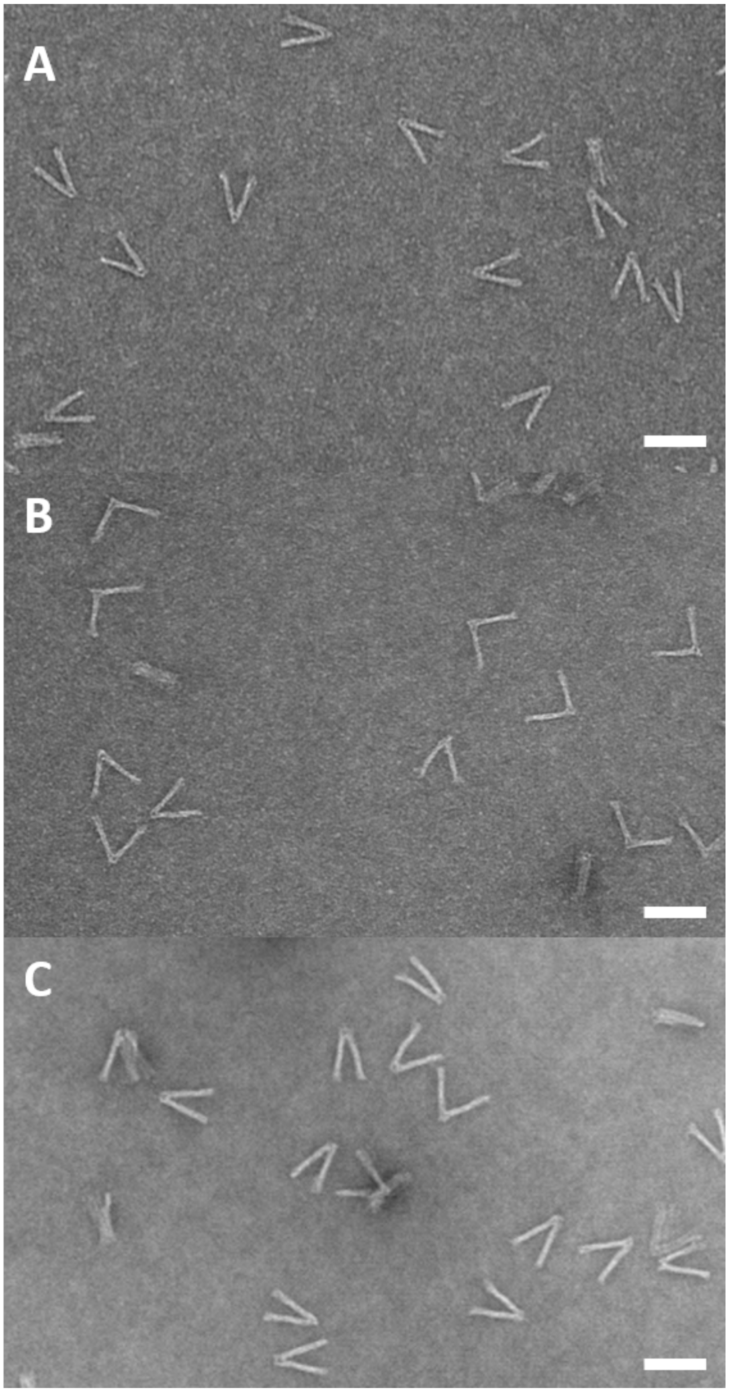

Figure S16 Representative TEM images of nDFS.B (A) Folded Closed (B) Toggled Open (C) Toggled Closed (Scale bars 100 nm). The toggling protocol is described in Methods of the main text.

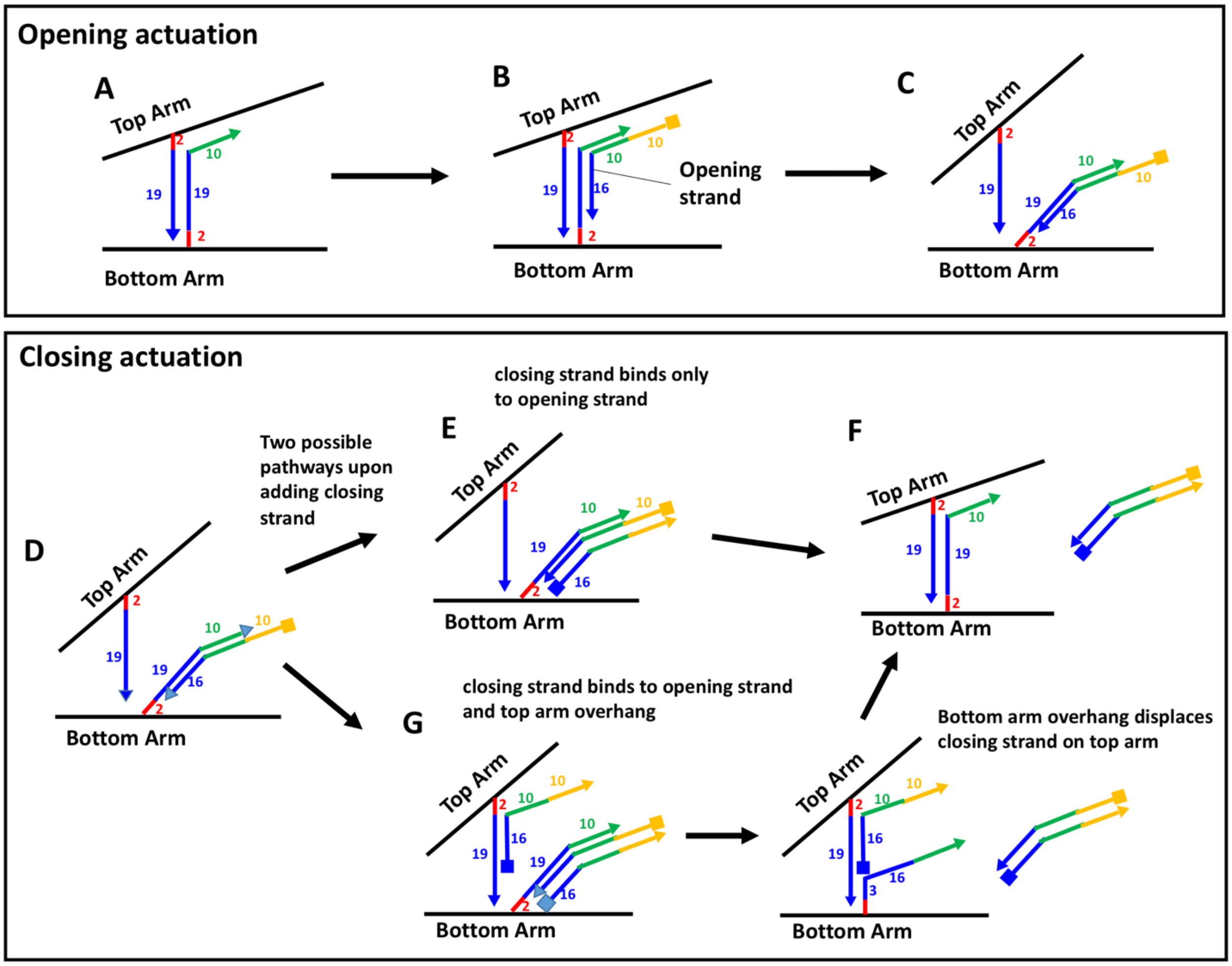

Figure S17 Toggling the nDFS by actuating the strut. The strut overhangs are shown in terms of each domain in a different color with length labels (unit: nt). For opening the nDFS: (A) The initial state is Folded Closed. The top strut overhang and bottom strut overhang form a 19 bp duplex (blue). The bottom strut has an additional 10 nt domain as toehold (green). The 2 thymine bases (red) are introduced between the arm and strut to facilitate 19 bp binding. (B) The opening strand is introduced to bind and displace the bottom strut. The opening strand contains an additional 10nt domain to serve as a toehold for closing (orange). (C) The 19 bp binding is released and nDFS toggles to the open state. For closing the nDFS: (D) The initial state is *Toggled Open*. When the closing strand is introduced, the actuation can follow two possible pathways. (E) The closing strand is introduced to bind and displace the opening strand. (F) The top strut and bottom strut re-form the 19 bp strut duplex, and the angular motion of nDFS is constrained again. (G) The closing strand can also bind to the top strut to form a 16 bp duplex. Since this duplex is only 16bp, it can be displaced by the bottom strut overhang. Excessive closing strand could increase the probability of this state and experiment (Figure S18) suggested inversely proportional relationship between toggling efficiency and closing strand concentration.

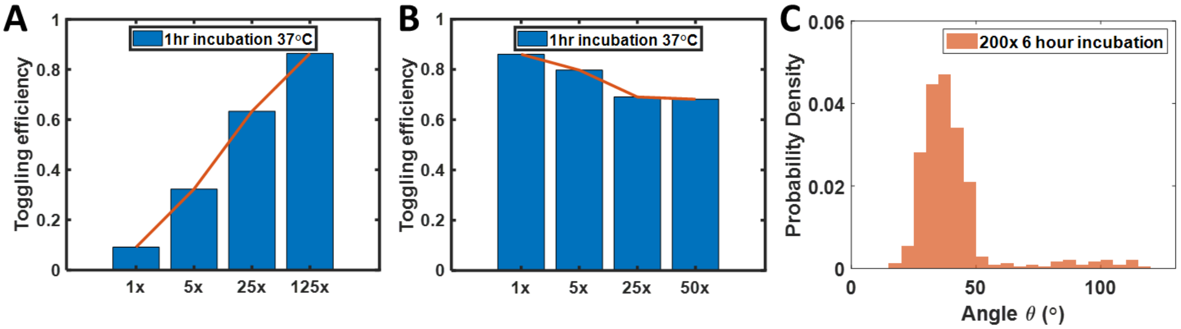

Figure S18 Toggling efficiency with varying displacement staple concentrations. A) The transition from Folded Closed to Toggled Open. B) The transition from Folded Open to Toggled Closed. The x-axis shows the excess ratio of the actuating strand to nDFS. C) Higher concentraion of closing strands can achieve high toggling efficiency (92%) if using 6 hour incubation time (closing strand at molar concentration 200 fold excess to nDFS).

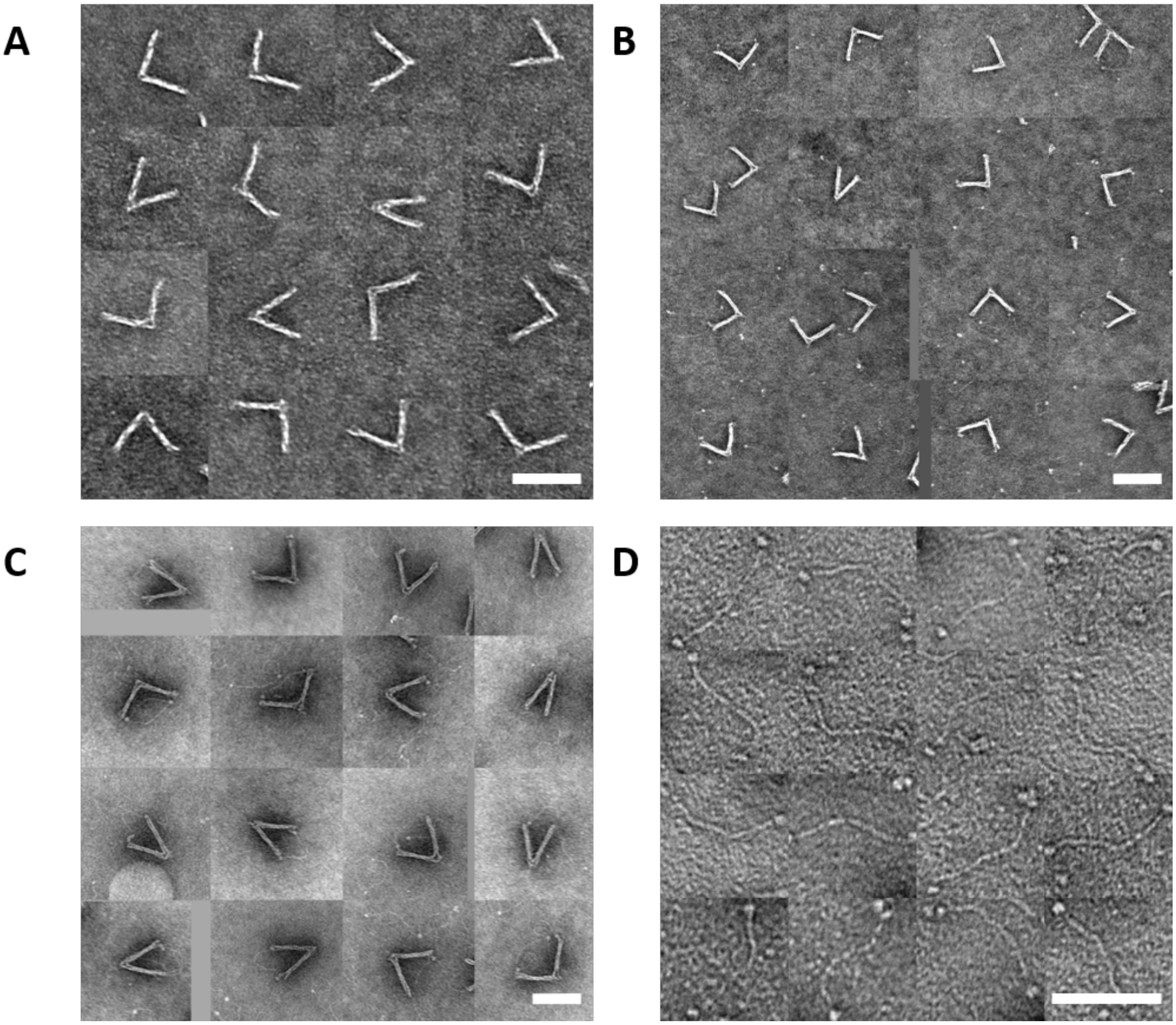

Figure S19 Representative TEM image galleries of structures within the compressive force workflow. TEM images of (A) *Folded Open* nDFS.B structures. (B) *Folded Open* nDFS.B structures with 249bp dsDNA (C) *Toggled Closed* nDFS.B structures with 249bp dsDNA. (D) Free 249bp dsDNA. (Scale bars 70 nm).

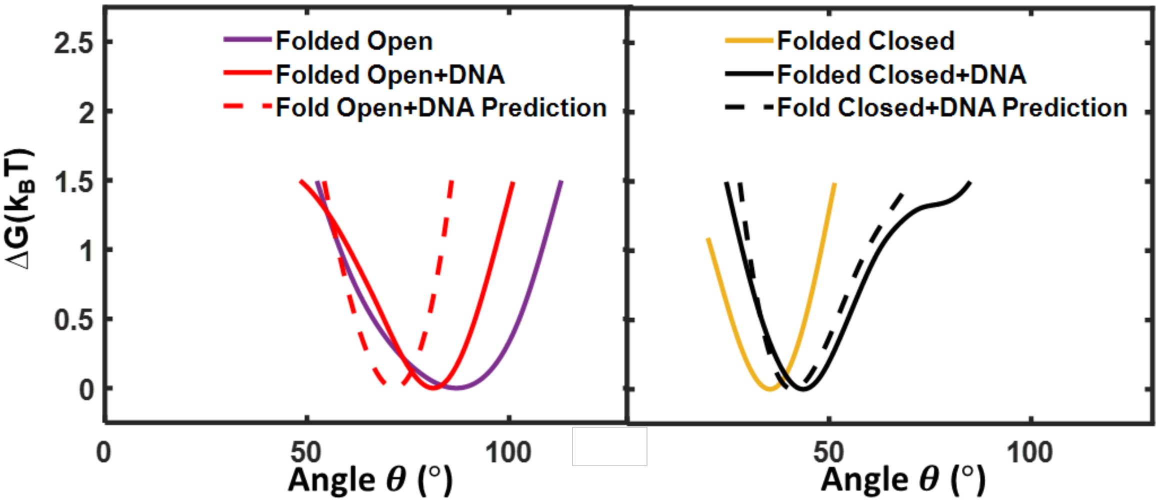

Figure S20 Free energy landscape comparison for open and closed nDFS both in the presence and absence of dsDNA.

Section 2: Fraying may explain discrepancy between experimental and predicted open hinge distributions

Although we model the hinge arms as a rigid body, both simulations and experimental data suggest that the bending of the hinge arms may not be negligible (Figure S21A). Additionally, Zhou et al (4) find that bending of DNA origami structures can be calculated using Euler-Bernoulli beam theory, and that the ends of structures with less crossover connections between helices are particularly susceptible to bending. oxDNA simulations of our own hinge also suggest qualitatively that fraying at the ends of the hinge arms for which there is no crosslinking may not be negligible. Accordingly, we first estimate bending stiffness by modelling the inner sections of the hinge arm for which there is crosslinking (see Figure S22), as Euler-Bernoulli beams following Zhou et al. (4). These sections consist of the inner 42 and 47 nm of the top and bottom arms, respectively, and are denoted as L_{bulk} . In our case, the bending stiffness of these sections is given by

$$BS_{bulk} = E_{dsDNA} I_{cs\_y} \tag{S2}$$

where E_{dsDNA} is the Young's modulus of DNA, and I_{cs_y} is the area moment of inertia of the cross section of the hinge arm. The Young's modulus is given by

$$E_{dsDNA} = \frac{L_p k_B T}{I_{DNA}} \tag{S3}$$

where L_p = 50 nm is the persistence length of dsDNA and I_{DNA} is the area moment of inertia of dsDNA.$I_{DNA} = \frac{\pi D^4}{64}$, where D = 2.5 nm is the diameter of dsDNA. For these hinges,

$$I_{cs\_y} = 16(I_{DNA} + (\frac{\pi D^2}{4})D^2) + 4I_{DNA} \tag{S4}$$

Combining these equations gives a bending stiffness of $BS_{bulk} \approx 5.6 \times 10^4 \text{pN} \cdot \text{nm}^2$. Now, in order to account for the lack of cross-linking at the end of the hinge, we model the final 19 nm and 14 nm of the top and bottom hinge arms, respectively, as beams made up of two dsDNAs, and denote this distance L_{fray} (i.e. $L_{fray} + L_{bulk} = L = 61$ nm, which is the total arm length). The bending stiffness in this case is given by

$$BS_{fray} = 2E_{dsDNA}I_{DNA} = 410\text{pN} \cdot \text{nm}^2 \quad \text{(S5)}$$

In order to calculate deflection given the bending stiffness, we follow Zhou by writing the beam slope angle $\theta(s)$ in terms of the inertial moment $M(s)$

$$\frac{d\theta(s)}{ds} = \frac{M(s)}{EI} \quad \text{(S6)}$$

where s is the coordinate along the hinge arm. For the geometry of our system,

$$\frac{d^2\theta(s)}{ds^2} = \frac{F_x}{EI}\cos\left[\theta(s)\right] \quad \text{(S7)}$$

where s is the coordinate along the hinge arm. When $s = 0$, the slope is defined to be $\theta(0) = 0$. Approximating the end of the beam, $s = L$, as the point at which force is applied, the moment $M(L) = 0$ and therefore $\theta'(L) = 0$. F_x (~ 0.9 pN) is the force exerted on the ends of the hinge by the DNA and connection, and it is approximated using the similar approach as discussed in Section 1.2. Solving this differential equation numerically with the indicated boundary conditions and integrating the infinitesimal deflection distance $\sin[\theta(s)]\, ds$ from $s = 0$ to $s = L$ gives the overall deflection for a single arm. The total deflection is taken to be the sum of deflections of the bulk beam (i.e. the integral is performed with BS_{bulk}) and the frayed end (i.e. the integral is performed with BS_{fray}). Explicitly, the total deflection is

$$w = \int_0^{L_{bulk}} \sin(\theta_{bulk}(s))ds + \int_0^{L_{fray}} \sin(\theta_{fray}(s))ds \quad \text{(S8)}$$

Note that we assume here that the force is acting on the end of the beam in both the bulk beam calculation and the frayed end calculation, but that this is only strictly true in the frayed end case (in fact the frayed end is acting directly on the bulk of the beam). Since we are only interested in an order of magnitude estimate, however, this small geometric approximation is reasonable.

The pieces of this calculation are shown schematically in Figure S21B, and the calculation predicts a deflection of on the order of 10 nm for the calculated force in the open state. This is on the order of the observed discrepancy: the peak of the experimental data is 82.5°, corresponding to an arm to an arm separation of 80 nm. The peak of the model prediction is at 72.5°, which corresponds to an arm separation of 72 nm. Therefore, the predicted fraying shift and observed discrepancy between model and prediction are both on the order of 10 nm, and we conclude that fraying is a reasonable possible explanation for this discrepancy. It is important to note, however, that we would not expect to see a fraying effect in the compressed case. Since the connections are on the inside of the hinge arms, in this case frayed ends would need to push through the bulk of the hinge, which is unlikely. Therefore, in the compressed case we assume there is no fraying on the ends, and only calculate the bulk hinge arm deflection. For a compression force of 0.3 pN with the equations given above, we predict a deflection of only about 1 nm.

The fraying is time-dependent although the timescale for those local fluctuations is much faster than any experimentally observable timescale. All hinges are expected to behave similarly with respect to the fraying as it is due to a reduction in cross-overs between helices at the end of the bundles and not due to some defect in the structure. This is difficult to avoid and is a common characteristic observed in DNA origami structures(5)(6).

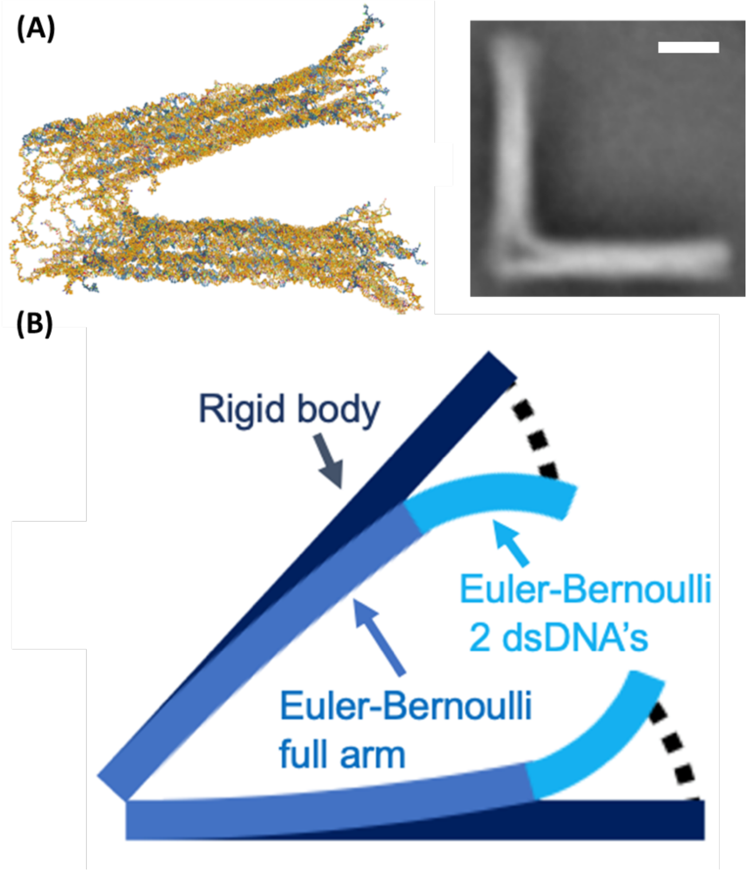

Figure S21 Fraying of nDFS. (A) Left: OxDNA simulation of the nDFS.A. The ends of the arm exhibit fraying. Right: Averaged TEM image for nDFS.B by using 60 similar angle samples also suggests fraying at the ends of hinge arms. (B) Schematic diagram of the beam model used to perform an approximate bending calculation. (Scale bars 70 nm).

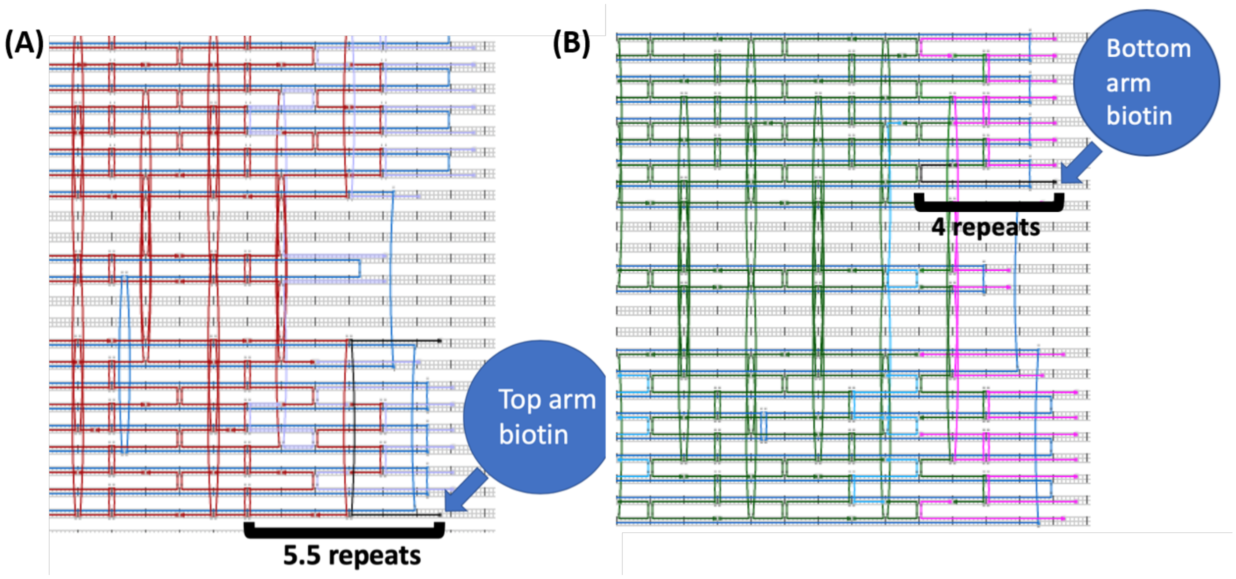

Figure S22 caDNAno design of ends of nDFS arms. (A) caDNAno schematic of the top arm with biotin location indicated. There are 5.5 helical repeats without cross-overs that connect several helices together, thereby effectively coupling the bundle cross-section. (B) caDNAno schematic of the bottom arm with

biotin location indicated. There are 4 helical repeats without cross-overs that effectively couple the entire cross-section.

Section 3: Solving the Elastica Model for Post-buckling Large Compression

In the prediction of DNA conformation in nDFS, the dsDNA is modeled as a Euler-Bernoulli beam (7). Figure S23 shows the beam in a post-buckling state with horizontal load P. Due to the symmetry of the problem, the beam only represents half of the dsDNA molecule with the fixed displacement and slope boundary condition at the Y axis ensuring the appropriate symmetry.

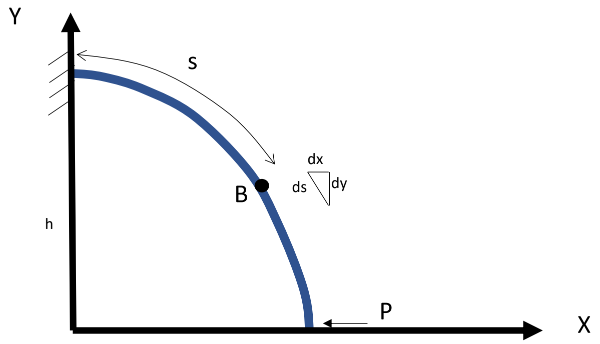

Figure S23 Beam deflection under horizontal load P

For any point B on the beam, the moment is

$$M(x,y) = -Py = \frac{EI}{r} \tag{S9}$$

where r is the radius of curvature. Define $k^2 = (\frac{P}{EI})$ and expand curvature, then

$$y = -\frac{\frac{d^2y}{dx^2}}{k^2[1 + (\frac{dy}{dx})^2]^{3/2}} \tag{S10}$$

Integrating with respect to x and with the fact $[1 + (\frac{dy}{dx})^2]^{1/2} = \frac{1}{\cos\theta}$

$$y^2 = \frac{2}{k^2}\cos\theta + C \tag{S11}$$

where C is the constant from integration. Define h as the distance from the origin to beam fixed point on the Y axis; then $h = (\frac{2}{k^2} + C)^{\frac{1}{2}}$.

Define $p = \frac{hk}{2}$ and $\sin\phi = \frac{\sin(\theta/2)}{p}$, by implementing trigonometric identities, it can be found:

$$y = h\cos\phi \tag{S12}$$

$$dy = -h\sin\phi\, d\phi \tag{S13}$$

Note that $y \in [0, h]$, $\phi \in [0, \frac{\pi}{2}]$.

Recall $\frac{dy}{ds} = \sin\theta$ and substituting dy from Eq. (S13) yields

$$ds = -\frac{hd\phi}{2p(1-p^2\sin^2\phi)^{\frac{1}{2}}} = -\frac{d\phi}{k(1-p^2\sin^2\phi)^{\frac{1}{2}}} \quad \text{(S14)}$$

The total length of the beam (or half-length of DNA)

$$L_b = \frac{1}{k}\int_0^{\frac{\pi}{2}} \frac{-d\phi}{(1-p^2\sin^2\phi)^{\frac{1}{2}}} \quad \text{(S15)}$$

Since the length of DNA is known to be 249*0.34/2=42.3 nm (note we are just dealing with half of the DNA due to symmetry), the variable p can be inversely determined numerically given a loading force P. The coordinates of the beam are:

$$y = \frac{2p\cos\phi}{k} \quad \text{(S16)}$$

$$x = \int_0^{\frac{\pi}{2}} dx = \int_0^{\frac{\pi}{2}} \cos\theta\, ds = \int_0^{\frac{\pi}{2}} \frac{(1-2p^2\sin^2\phi)}{k(1-p^2\sin^2\phi)^{\frac{1}{2}}} d\phi \quad \text{(S17)}$$

Explicitly determining the loading force can be very difficult since the real DNA curve does not necessarily match the Elastica solution. Therefore practically, we can test a range of loading forces and the corresponding DNA shape. By comparing these plots with the experimental DNA curve, the loading force can be inversely determined. If we use 50 nm as persistence length, the compression force from nDFS.B would be 0.4±0.1 pN, where the error bar is determined from the standard deviation of the end-to-end distance of dsDNA (19nm) after compression.

Since the persistence length of dsDNA might be influenced by salt conditions (3), we also checked different persistence lengths L_p from 40 nm to 60 nm (Figure S24) and found the compression force to have a linear-like dependence on the persistence length. However, the force is still in the range of 0.1-1 pN.

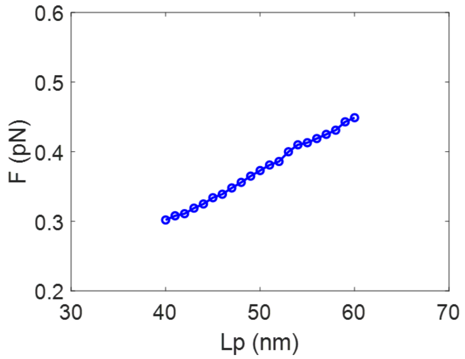

Figure S24 Influence of dsDNA persistence length on compression force.

Table S7 Basic parameters used in the Elastica model

Symbol	Parameter	Value
L_b	Length of half DNA	42.3 nm
L_p	Persistence Length	50 nm
k_B	Boltzmann constant	$1.38\times10^{-23} m^2kgs^{-2}K^{-1}$
T	Temperature	Room temperature (300 K)

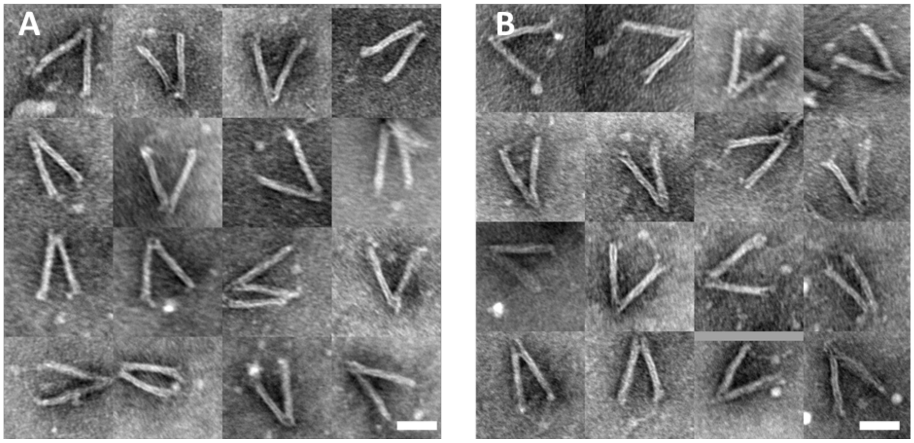

Figure S 25 Representative TEM image galleries of structures with nucleosomes. (A) nDFS.C-35 (B) nDFS.B. (Scale Bars 50 nm)

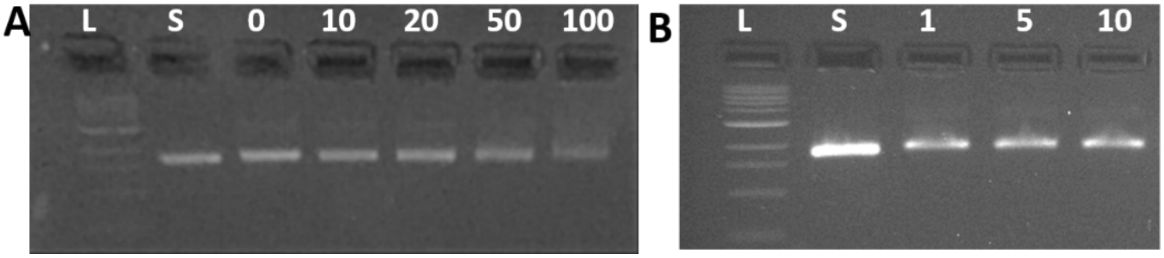

Figure S26 nDFS stability in serum and varying ion conditions. (A) nDFS in various concentration FBS buffer conditions in a background of RPMI for 4 hour incubation at 37°C. L: 1kb Ladder; S: 8064 Scaffold. Numbers are FBS percentage. (B) nDFS in buffer condition 50 mM Tris, 200 mM NaCl, and [1,5,10] mM $MgCl_2$ for 4 hour incubation at 37°C.

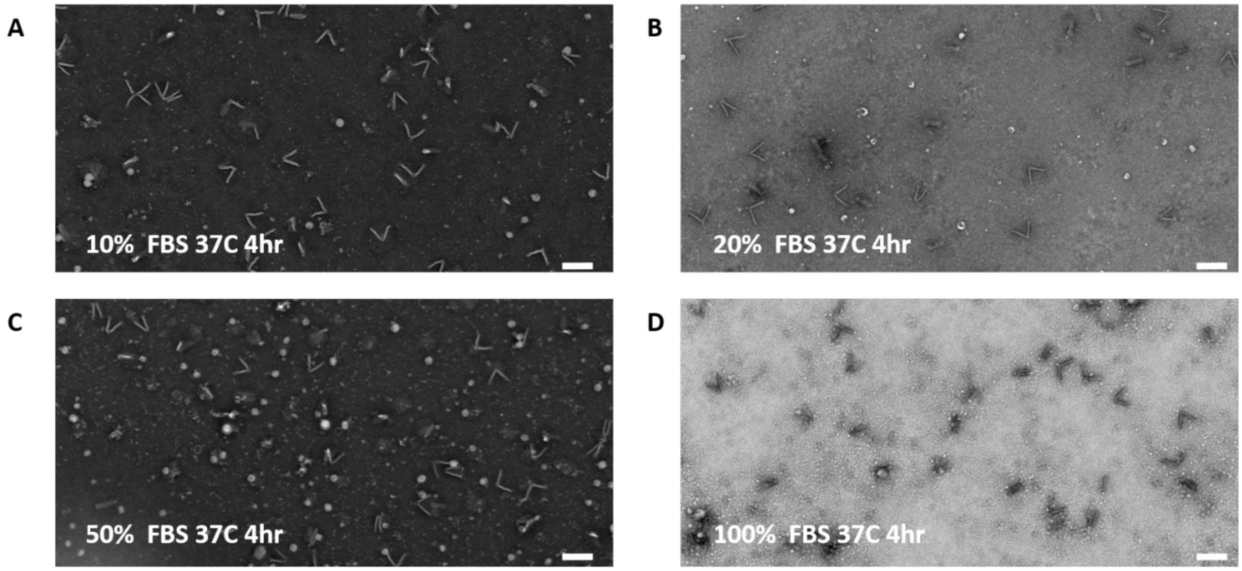

Figure S27 FBS TEM Images. Typical TEM images of nDFS.A after PEG purification, 4-hour FBS incubation. Structures were incubated in (A) 10% FBS , (B) 20% FBS, (C) 50%FBS, (D) 100% FBS. (Scale bars 100 nm).

Reference

1. Wilhelm,J. and Frey,E. (1996) Radial Distribution Function of Semiflexible Polymers. *Phys. Rev. Lett.*, **77**, 2581–2584.
2. Šulc,P., Romano,F., Ouldridge,T.E., Rovigatti,L., Doye,J.P.K. and Louis,A.A. (2012) Sequence-dependent thermodynamics of a coarse-grained DNA model. *J. Chem. Phys.*, **137**.
3. Baumann,C.G., Smith,S.B., Bloomfield,V.A. and Bustamante,C. (1997) Ionic effects on the elasticity of single DNA molecules. *Proc. Natl. Acad. Sci. U. S. A.*, **94**, 6185–6190.
4. Zhou,L., Marras,A.E., Su,H.J. and Castro,C.E. (2014) DNA origami compliant nanostructures with tunable mechanical properties. *ACS Nano*, **8**, 27–34.
5. Kim,D.N., Kilchherr,F., Dietz,H. and Bathe,M. (2012) Quantitative prediction of 3D solution shape and flexibility of nucleic acid nanostructures. *Nucleic Acids Res.*, **40**, 2862–2868.
6. Huang,C.M., Kucinic,A., Le,J. V., Castro,C.E. and Su,H.J. (2019) Uncertainty quantification of a DNA origami mechanism using a coarse-grained model and kinematic variance analysis. *Nanoscale*, **11**, 1647–1660.
7. Frisch-Fay,R. (1962) Flexible Bars Butterworths.
